# Moderating effect of mode of delivery on the genetics of intelligence: Explorative genome‐wide analyses in ALSPAC

**DOI:** 10.1002/brb3.1144

**Published:** 2018-10-31

**Authors:** Dinka Smajlagić, Kaya Kvarme Jacobsen, Craig Myrum, Jan Haavik, Stefan Johansson, Tetyana Zayats

**Affiliations:** ^1^ Department of Clinical Science, KG Jebsen Center for Neuropsychiatric Disorders University of Bergen Bergen Norway; ^2^ Center for Medical Genetics and Molecular Medicine Haukeland University Hospital Bergen Norway; ^3^ Department of Biomedicine, KG Jebsen Center for Neuropsychiatric Disorders University of Bergen Bergen Norway

**Keywords:** Avon Longitudinal Study of Parents and Children, glutamate, intelligence, mode of delivery

## Abstract

**Introduction:**

Intelligence is a core construct of individual differences in cognitive abilities and a strong predictor of important life outcomes. Within recent years, rates of cesarean section have substantially increased globally, though little is known about its effect on neurodevelopmental trajectories. Thus, we aimed to investigate the influence of delivery by cesarean section on the genetics of intelligence in children.

**Methods:**

Participants were recruited through the Avon Longitudinal Study of Parents and Children (ALSPAC). Intelligence was measured by the Wechsler Intelligence Scale for Children (WISC). Genotyping was performed using the Illumina Human Hap 550 quad genome‐wide SNP genotyping platform and was followed by imputation using MACH software. Genome‐wide interaction analyses were conducted using linear regression.

**Results:**

A total of 2,421 children and 2,141,747 SNPs were subjected to the genome‐wide interaction analyses. No variant reached genome‐wide significance. The strongest interaction was observed at rs17800861 in the GRIN2A gene (*β* = −3.43, 95% CI = −4.74 to −2.12, *p* = 2.98E−07). This variant is predicted to be located within active chromatin compartments in the hippocampus and may influence binding of the NF‐kappaB transcription factor.

**Conclusions:**

Our results may indicate that mode of delivery might have a moderating effect on genetic disposition of intelligence in children. Studies of considerable sizes (>10,000) are likely required to more robustly detect variants governing such interaction. In summary, the presented findings prompt the need for further studies aimed at increasing our understanding of effects various modes of delivery may have on health outcomes in children.

## 1 INTRODUCTION

Intelligence is a core construct of individual differences in cognitive abilities. Measured intelligence is a strong predictor of important life outcomes, such as mental and physical health and mortality (Deary, Weiss, & Batty, 2010). It is well established that intelligence is a highly heritable behavioral trait (Davies et al., 2012), with heritability increasing from childhood to adulthood, while maintaining genetic stability across a lifespan (Plomin & Deary, 2014). Nonetheless, the extent and nature of such genetic influence is still not clear (Visscher, Hill, & Wray, 2008) and warrants further investigation.

Intelligence captures a broad scope of cognitive abilities and can be enumerated by measures of vocabulary or verbal IQ (VIQ) and matrix reasoning or performance IQ (PIQ) that can be combined into a full scale IQ (FSIQ; David, 1991). Neurobiological studies on intelligence have linked it to brain structure and functioning (Gray & Thompson, 2004; Song et al., 2008). For example, it has been shown that VIQ and PIQ capture different aspects of intelligence—both behaviorally and biologically (Nisbett et al., 2012). VIQ includes knowledge accumulated throughout life (e.g., vocabulary), determined by education and experience (crystallized intelligence), while PIQ reflects problem solving and reasoning abilities (e.g., matrix reasoning) that has little reliance on stored knowledge or learning (fluid intelligence; Toga & Thompson, 2005).

The heritability of intelligence is hypothesized to be influenced by many genes of small effects, and it increases from approximately 20% in early childhood to 60% in adulthood (Haworth et al., 2010). However, despite this increase, the same genes are believed to influence intelligence during the life course (genetic stability; Plomin & Deary, 2014). To date, the catalogue of genome‐wide association (GWA) studies (https://www.ebi.ac.uk/gwas/home, accessed on May 24, 2018) reports nine studies, using “intelligence” as a search word (Benyamin et al., 2014; Butcher, Davis, Craig, & Plomin, 2008; Davies et al., 2012; Davis et al., 2010; Gialluisi et al., 2014; Hill et al., 2018; Kirkpatrick, Mcgue, Iacono, Miller, & Basu, 2014; Loo et al., 2012; Sniekers et al., 2017). The biggest study among those was performed on 248,482 individuals, using combined data for intelligence and correlated phenotypes (Hill et al., 2018). The authors reported 187 genome‐wide significant independent loci, showing that the combined effects of common single nucleotide polymorphisms (SNPs) across the genome explain only 25.44% of phenotypic variation in intelligence (Hill et al., 2018).

Apart from genetics, the environment also plays a substantial role in the variability of intelligence. Genes can affect intelligence directly depending on the environment (gene–environment interaction) or indirectly through gene–environment correlation (Gray & Thompson, 2004). The increase in heritability of intelligence, assuming genetic stability, might occur due to contributions from genetic amplification, where genetic influences are amplified in a selected environment (Plomin & Deary, 2014). The examinations of environmental influences on the genetic underpinnings of human intelligence are, therefore, an important area of research.

Studies on the environmental effects on intelligence have more commonly been done within the framework of heritability estimates in twin studies (Deary, Johnson, & Houlihan, 2009), while molecular gene–environment interaction analyses are sparse. Interaction analyses may provide an estimate of the extent to which a specific environment affects the variability in intelligence, given a certain genetic predisposition. Such estimates may help our understanding of intelligence's etiology as well as provide insights into how intelligence may be affected by changes in our day‐to‐day lives, and to, eventually, inform healthcare practices.

Delivery by cesarean section (DCS) refers to giving birth through surgical incisions in the mother's abdomen and uterus as opposed to vaginal delivery (VD), occurring via contractions of uterine muscles. DCS can be a life‐saving procedure when either the mother or the baby is at high risk of adverse outcomes (Black, Bhattacharya, Philip, Norman, & McLernon, 2015). As cesarean sections performed on mother's demand have become increasingly common in recent years (Christilaw, 2006; D'Souza, 2013; Kwee, Cohlen, Kanhai, Bruinse, & Visser, 2004; Lavender, Hofmeyr, Neilson, Kingdon, & Gyte, 2014), the number of DCSs has substantially risen worldwide (Belizán, Althabe, & Cafferata, 2007; Villar et al., 2006), although the short‐ and long‐term effects of both DCS and VD on health outcomes in children remain unclear.

So far, epidemiological studies suggest that DCS may present increased risk for developing wheezing and asthma (Magnus et al., 2011; Thavagnanam, Fleming, Bromley, Shields, & Cardwell, 2008), childhood obesity (Kuhle, Tong, & Woolcott, 2015), and type 1 diabetes mellitus (Black et al., 2015; Cardwell et al., 2008). Experimental data also implicate the lack of VD in altered immune function, stress response, and epigenetic modifications in children (Ronca, Abel, Ronan, & Alberts, 2009; Round & Mazmanian, 2009; Salminen, Gibson, McCartney, & Isolauri, 2004; Schlinzig, Johansson, Gunnar, Ekström, & Norman, 2009).

The effect of delivery method has also been studied in relation to development of the nervous system and related phenotypes. It has been recently shown that VD can be implicated in gene expression involved in neuroprotection, synaptogenesis, and neuronal differentiation in the hippocampus—a brain region important for memory formation (Seli & Horvath, 2013; Simon‐Areces et al., 2012; Varela, Schwartz, & Horvath, 2016). Since intelligence has been linked to working memory (Kane & Engle, 2002; Kyllonen & Christal, 1990) and activity‐dependent synaptic plasticity in the hippocampus (Morris et al., 2003; Neves, Cooke, & Bliss, 2008), developmental disturbances of either could potentially affect intelligence, which in turn is associated with a number of neurodevelopmental disorders (Kendler, Ohlsson, Sundquist, & Sundquist, 2015; Kuntsi et al., 2004). DCS has also been implicated in long‐term changes in the dopaminergic system (Boksa, Zhang, & Bestawros, 2002; Brake, Noel, Boksa, & Gratton, 1997; El‐Khodor & Boksa, 1997; Vaillancourt & Boksa, 1998), believed to play role in the pathomechanisms of several neuropsychiatric conditions (Bassett, Chow, Waterworth, & Linda, 2008; Grace, 2012; Homberg et al., 2016; Martelle et al., 2016). Indeed, DCS has been linked to neurodevelopmental impairments (Wilson‐Costello et al., 2007), special educational needs in later life (Kapellou, 2011; Mackay, Smith, Dobbie, & Pell, 2010), autism (Curran et al., 2015; Emberti Gialloreti, Benvenuto, Benassi, & Curatolo, 2014; Gregory et al., 2009), attention‐deficit/hyperactivity disorder (Curran et al., 2016; Sucksdorff et al., 2018), and obsessive–compulsive disorder (Vasconcelos et al., 2007). Children delivered by cesarean section may also face more emotional disturbances, withdrawal, and sleep problems and display a high number of internalizing problems during preschool ages (Kelmanson, 2013).

Taken together, the method of delivery has been reported to be associated with a number of neurodevelopmental processes and disorders of the brain, all of which have also been linked to intelligence. Given the high heritability and substantial environmental effect on the variability of intelligence, we aimed to investigate a possible interplay between genetic factors and mode of delivery on the development of intelligence in children. We performed a genome‐wide examination of moderating effect of mode of delivery on the genetics of intelligence (gene × environment interaction) in a sample of 2,421 children aged 8.5 years. We then examined the robustness of the interaction by accounting for potential confounding perinatal factors, such as gestational age and Apgar score.

## 2 MATERIALS AND METHODS

### 2.1 Participants

The participants of this study were recruited through the Avon Longitudinal Study of Parents and Children (ALSPAC; RRID:SCR_007260), also known as “Children of the 90s” (Boyd et al., 2013). All pregnant women living in the Avon County with estimated delivery dates between April 1, 1991, and December 31, 1992, were eligible to participate. Of the original 14,541 pregnancies, 13,988 children were alive at one year of age. An additional 713 children were enrolled after age seven, resulting in a total sample of 14,701 children. The data were collected from 14,009 participants (self‐reported or provided by biological mother/primary caregiver). These mother–child pairs have been followed for over 20 years, generating an immense amount of data through biological samples, measurements, and questionnaires. The study website contains details of all the data that is available through a fully searchable data dictionary which can be obtained here: https://www.bris.ac.uk/alspac/researchers/data-access. Phenotype‐matched genotype data were available for up to 6,832 children, depending on the variables. Ethical approval for the study was obtained from the ALSPAC's own Ethics and Law Committee and the Local Research Ethics Committees in the UK (Bristol and Weston Health Authority: E1808 Children of the Nineties: Avon Longitudinal Study of Pregnancy and Childhood (ALSPAC); Southmead Health Authority: 49/89 Children of the Nineties—“ALSPAC”; Frenchay Health Authority: 90/8 Children of the Nineties; United Bristol Healthcare Trust: E4445 ALSPAC Focus at Eight; Southmead (North Bristol Trust): Project 084/99 ALSPAC Assessments at Age Eight; Frenchay (North Bristol Trust): Project 99/42 Avon Longitudinal Study of Parents and Children (ALSPAC). Assessments at Age Eight (ALSPAC Focus at Eight); Weston Area Health Trust: E177 Avon Longitudinal Study of Parents and Children (ALSPAC). Assessments at Age Eight).

### 2.2 Genotyping and quality control (QC)

ALSPAC participants’ DNA was extracted from whole blood or buccal swab samples and prepared for genotyping using standard protocols. A total of 9,912 samples were genotyped using the Illumina Human Hap 550 quad genome‐wide SNP genotyping platform, and quality control was performed by ALSPAC as described at https://www.bristol.ac.uk/media-library/sites/alspac/migrated/documents/gwas-data-generation.pdf. In short, SNPs with a minor allele frequency (MAF) <1%, with a call rate <95%, and out of Hardy–Weinberg equilibrium (*p* < 5.00E−07) were removed. The resulting data set consisted of 8,365 individuals.

Known autosomal variants were imputed by ALSPAC with MACH version 1.0.16 (RRID:SCR_007260) Markov Chain Haplotyping software (Li, Willer, Ding, Scheet, & Abecasis, 2010; Li, Willer, Sanna, & Abecasis, 2009), using the Centre d'Etude du Polymorphisme Humain (CEPH) individuals from phase 2 of the HapMap Project (HG18; RRID:SCR_002846) as a reference set (release 22). Imputation of X chromosomal SNPs was done with MiniMac version 4.43 (RRID:SCR_009292; Howie, Fuchsberger, Stephens, Marchini, & Abecasis, 2012). Only SNPs with imputation quality estimates above 0.3 were included.

We performed additional quality control in PLINK software (RRID:SCR_001757), version 1.09 (Purcell et al., 2007), in the subset of individuals with available IQ measures to ensure that no SNPs or individuals had poor genotyping rates (<98%), no SNPs were rare (MAF <5%) or SNPs were out of Hardy–Weinberg equilibrium (*p* < 1.00E−06). In addition, we removed individuals revealing cryptic relatedness (PI_HAT>15%), excessive heterozygosity (outside the range of mean ± 3 standard deviations (*SD*)), incorrect sex assignment, and/or non‐European ancestry based on multidimensional scaling with HapMap data (phase 3).

### 2.3 Measure of intelligence and perinatal factors

Intelligence was assessed via the Wechsler Intelligence Scale for Children (WISC; Wechsler, Rust, & Golombok, 1992). Verbal intelligence quotient (VIQ) was measured based on five oral subtests: information, similarities, arithmetic, vocabulary, and comprehension. Performance IQ (PIQ) subtests included nonverbal problems: picture completion, coding, picture arrangement, block design, and object assembly. Full scale IQ (FSIQ) was calculated based on verbal and performance IQ scores. All IQ measures were subjected to outlier removal (outside the range of mean ± 3 *SD*), and normal distribution was ensured.

Perinatal factors have been reported to play a role in neuropsychiatric disorders where IQ is affected as well as to highly correlate with DCS (Guinchat et al., 2012; Halmøy, Klungsøyr, Skjærven, & Haavik, 2012; Sucksdorff et al., 2018). We, therefore, examined their possible confounding effect on the relationship between mode of delivery and the genetics of intelligence. Available perinatal factors included birth weight, birth length, Apgar score measured at 1 min, and gestational age. To avoid collinearity, we assessed correlation among all of the aforementioned factors as well as their correlation with mode of delivery. Factors revealing correlation above 30% were excluded from the analyses.

### 2.4 Genome‐wide interaction analyses

We explored the moderating effect of mode of delivery on the genetics of intelligence by performing linear regression analyses. We first constructed crude models with each of the IQ measurements as an outcome (three models in total), examining the main effects of SNPs, mode of delivery, and sex. We also included a two‐way interaction term between SNPs and mode of delivery. SNPs were tested assuming additive model in PLINK version 1.09 (Purcell et al., 2007).

As the effect of cesarean section may be confounded by other perinatal factors, the initial crude models were also examined adjusted for those factors. Since birth weight, birth length, and gestational age revealed high correlation (Supporting Information Table S1), only Apgar score and gestational age were included as covariates in the adjusted models to avoid collinearity. None of the models were adjusted for age as all participants were of the same age (8.5 years), but all models were adjusted for sex. Interaction analyses place a high parameter burden on samples of modest sizes, so cell counts of the most significant variants were examined. When those were below 5, the models were analyzed assuming a dominant genetic effect.

To correct for multiple testing, genome‐wide significance threshold was set at a *p*‐value of 5.00E−08 (Risch & Merikangas, 1996). Calculation of genomic control coefficient (lambda (*λ*)) and QQ plots were utilized to ensure integrity of the observed test statistics.

### 2.5 In silico exploration of the most significant SNPs

To assess possible functional relevance of our top findings, we performed in silico analyses of the most significant SNPs using HaploReg software (RRID:SCR_006796), version 4.1 (Ward & Kellis, 2012). HaploReg is a tool for exploring chromatin states and regulatory motif alterations among variants in the genome (https://archive.broadinstitute.org/mammals/haploreg/haploreg.php).

## 3 RESULTS

### 3.1 Subjects, genotyping, QC, and measure of intelligence and perinatal factors

Overall, 2,141,747 SNPs and 2,421 individuals passed all QC filters. The same number of individuals had all phenotypes available for the analyses. Table 1 summarizes the examined phenotypes, the number of participants, and the sex distribution by each IQ measure. The summary of the overall data processing, reflecting the number of participants depending on the availability of the IQ measure, genetic data, and performed quality controls is shown in Supporting Information Figure S1 (constructed in PRISMA (Liberati et al., 2009)). The distribution of IQ measures is presented in Supporting Information Figures S2–S4.

**Table 1:** Overview of the individuals and phenotypes included in this study

	Spontaneous delivery *N* = 1,902	Delivery by cesarean section *N* = 519	Difference *p*‐Value
Full scale IQ (mean (*SD*))	102.82 (19.54)	103.95 (20.34)	0.25^a^
Sex (female %)	52.05%	49.32%	0.29^b^
Apgar score at 1 min (mean (*SD*))	8.51 (1.14)	7.70 (2.10)	6.08E−12^c^
Gestational age (mean (*SD*))	39.46 (1.82)	38.69 (2.48)	6.71E−15^a^

a One‐way ANOVA.

b Chi‐squared test.

c Wilcoxon nonparametric test.

### 3.2 Genome‐wide interaction analyses

We did not observe any genome‐wide significant main effect at any SNP in any of our models. Furthermore, our interaction analyses did not reveal any genome‐wide significant findings (*p* < 5.00E−08) for any of the intelligence measures in the models examined.

**Figure 1:**
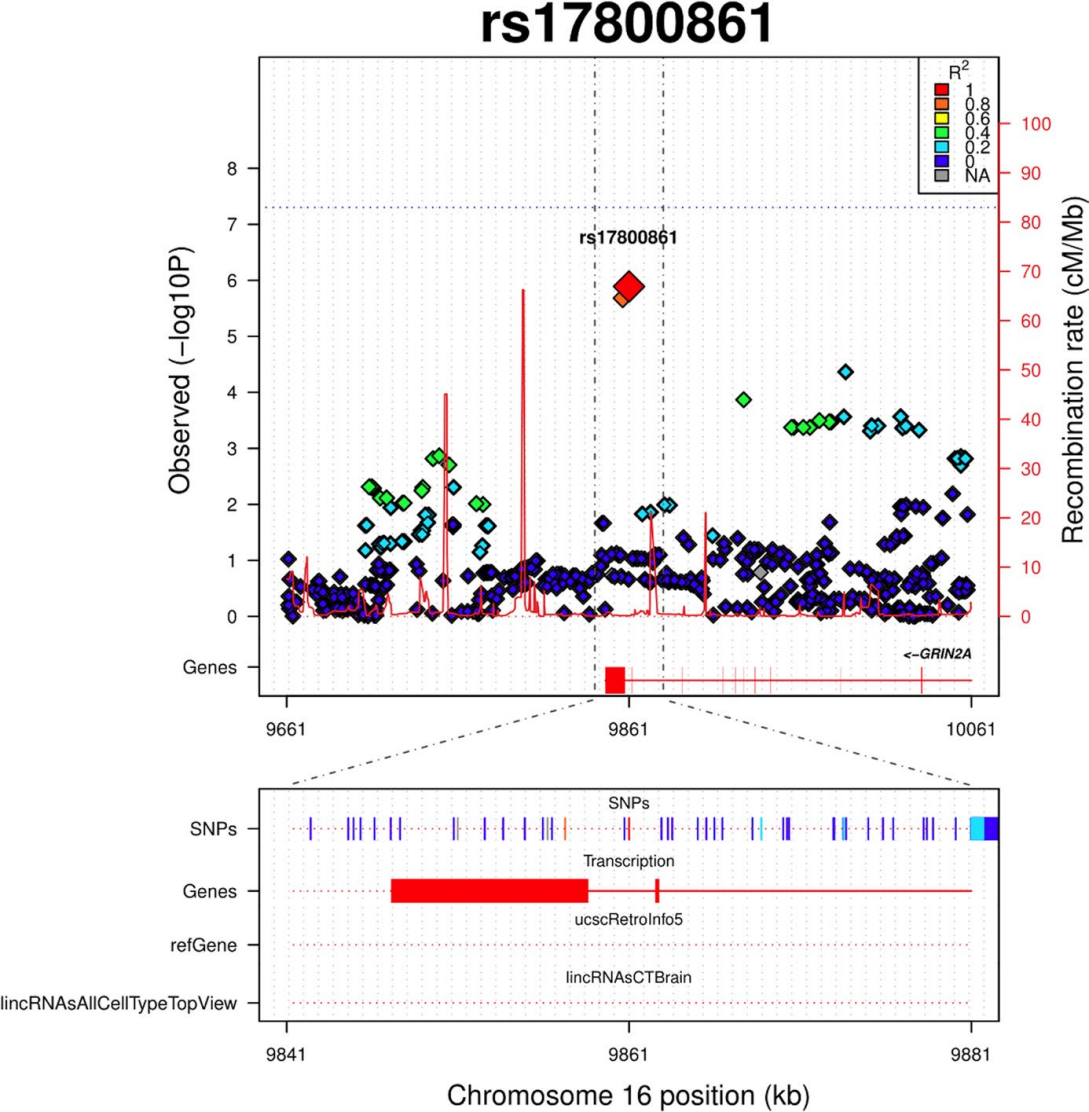
Regional plot of rs17800861. Variants are plotted by position on chromosome 16 against the observed interaction p‐values for FSIQ measure in the adjusted model. Local LD structure is reflected by estimated recombination rates from the HapMap CEU population (Utah residents with Northern and Western European ancestry plotted in red on the right side). The colors of the variants surrounding rs17800861 are reflecting their LD (according to pairwise *r* ^2^ values from the HapMap CEU population). “Genes” refers to protein‐coding genes in the presented region. “refGenes” refers to both protein‐coding and non‐protein‐coding genes reflecting the data from RefSeq UCSC tracks. “lincRNAsAllCellTypeTopView” reflects the data from the lncRNA UCSC tracks in brain tissue

Among the crude models, the most significant signals were observed at rs705670 for PIQ, rs17800861 for FSIQ, and rs1276529 for VIQ (Table 2). After adjusting for possible confounders, the strongest interaction was observed at rs17800861 for FSIQ in the intron of the *GRIN2A* (glutamate receptor, ionotropic *N*‐methyl‐d‐aspartate 2A gene (*β* = −3.43, 95% CI = −4.74 to −2.12, *p* = 2.98E−07; Table 2 and Figure 1). The second strongest interaction signal was observed for PIQ at rs705670 (*β* = 2.31, 95% CI = 1.43 to 3.19, *p* = 3.09E−07; Table 2 and Supporting Information Figures S5), an intronic variant within a long intergenic non‐protein‐coding RNA gene (*LINC01502*) located on chromosome 9. For VIQ, the strongest signal was noted at rs1276529 (*β* = −2.09, 95% CI = −2.91 to −1.26, *p* = 7.13E−07; Table 2 and Supporting Information Figures S6), an intergenic polymorphism on chromosome 6.

**Table 2:** Summary of the top three SNPs observed in this study

SNP	CHR	BP	Effect allele	Gene	MAF	Crude model			Adjusted model		
						*β*	CI	Interaction *p*‐value	*β*	CI	Interaction *p*‐value
Verbal IQ											
rs1276529	6	112,921,192	G	*RFPL4B*	0.32	−2.04	−2.86 to −1.22	1.10E−06	−2.09	−2.91 to −1.26	7.13E−07
rs1706066	6	112,923,618	G	*RFPL4B*	0.32	−2.04	−2.86 to −1.22	1.10E−06	−2.09	−2.91 to −1.26	7.13E−07
rs1276583	6	112,917,585	G	*RFPL4B*	0.35	−1.98	−2.79 to −1.18	1.33E−06	−1.99	−2.79 to −1.18	1.42E−06
Performance IQ											
rs705670	9	137,608,405	G	*LINC01502*	0.28	2.30	1.42 to 3.18	3.07E−07	2.31	1.43 to 3.19	3.09E−07
rs12552228	9	27,163,693	T	*TEK*	0.09	2.49	1.33 to 3.65	2.70E−05	2.79	1.61 to 3.97	3.87E−06
rs12554799	9	27,163,704	G	*TEK*	0.09	2.49	1.33 to 3.65	2.70E−05	2.79	1.61 to 3.97	3.87E−06
Full scale IQ											
rs17800861^a^	16	9,861,173	A	*GRIN2A*	0.11	−3.32	−4.63 to −2.01	7.22E−07	−3.43	−4.74 to −2.12	2.98E−07
rs12552228	9	27,163,693	T	*TEK*	0.09	2.44	1.33 to 3.55	1.81E−05	2.84	1.71 to 3.96	8.51E−07
rs12554799	9	27,163,704	G	*TEK*	0.09	2.44	1.33 to 3.55	1.81E−05	2.84	1.71 to 3.96	8.51E−07

a Dominant model.

**Figure 2:**
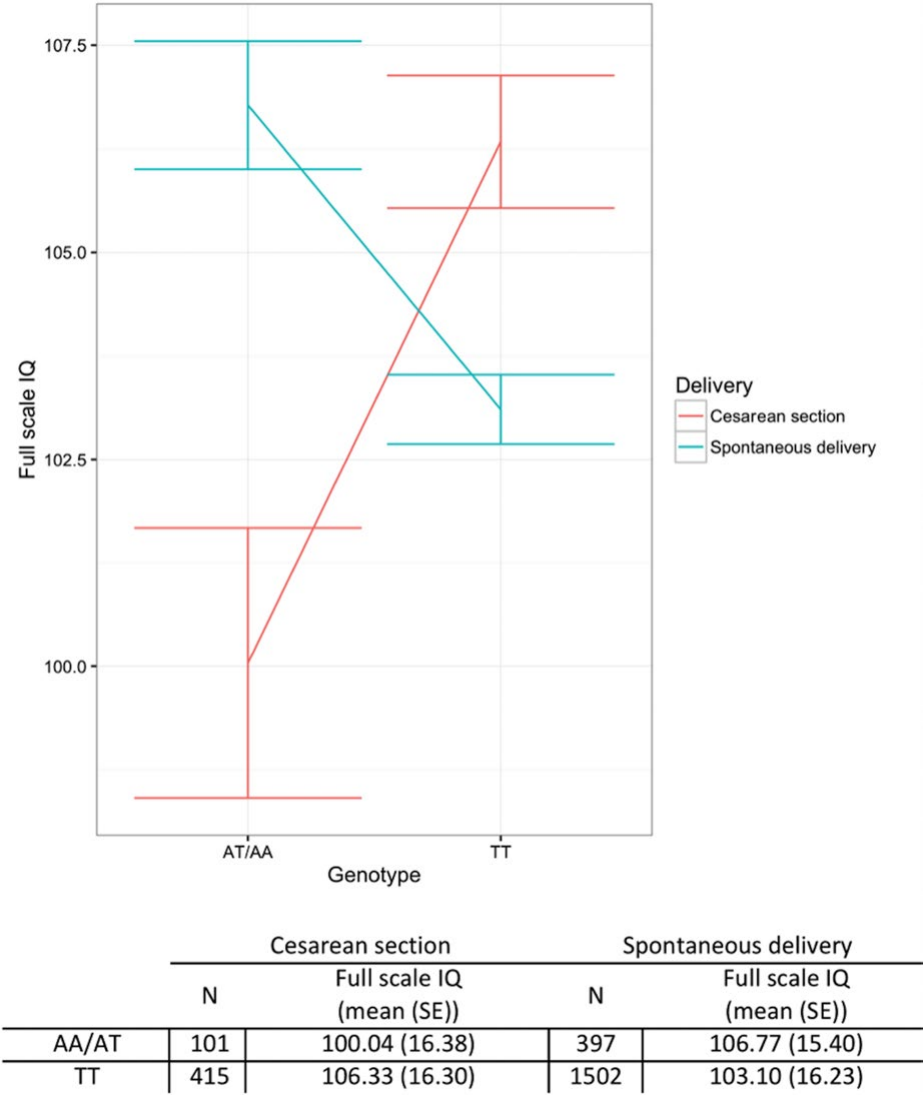
Interaction plot reflecting a moderating effect of delivery mode on the correlation between FSIQ scores and rs17800861. The *y*‐axis represents the FSIQ scores, while the *x*‐axis displays rs17800861 genotypes. Error bars represent the standard error of the FSIQ scores

The presence of at least one copy of the A allele at rs17800861 revealed association with lower FSIQ score in children delivered by cesarean section, but not in those delivered vaginally (Figure 2). Similar to rs17800861, the presence of at least one copy of the minor allele at rs1276529 was associated with lower VIQ scores only in children delivered by cesarean section (Supporting Information Figures S7). For rs705670, the presence of at least one copy of the minor allele was associated with higher PIQ scores in children delivered by cesarean section, but not in those delivered by VD (Supporting Information Figures S8). These results remained similar after accounting for other possible confounding perinatal factors (Table 2).

Supporting Information Tables S2–S4 present top interaction signals (*p* < 1.00E−04) observed in this study. The QQ plots are depicted in Supporting Information Figures S9–S11 and Manhattan plots in Supporting Information Figures S12–S14. *λ* is close to one in all three models.

### 3.3 In silico assessment of the most significant SNPs

Examination of the strongest variant, rs17800861, in HaploReg revealed that this SNP is predicted to be located within active chromatin compartments, having transcription enhancer properties in the hippocampus—an important brain region for memory (Morris et al., 2003). The neurons of hippocampus are also capable of neurogenesis, which may be affected by the method of delivery (Seli & Horvath, 2013). This SNP is also reported to alter the NF‐kappaB transcription factor binding motif (https://archive.broadinstitute.org/mammals/haploreg/haploreg.php). NF‐kappaB is a ubiquitous transcription factor, acting as a master switch for the expression of a number of genes involved in immune and inflammatory responses (Sieben, Franzoso, & Brown, 1994). No other SNPs displayed functional activity in the hippocampus, and no SNPs revealed expression quantitative trait locus (eQTL) activity.

## 4 DISCUSSION

The central hypothesis explored in this study was whether the method of delivery may moderate the genetic disposition of intelligence in children. While we did not observe any genome‐wide significant interactions (*p* < 5.00E−08), we did note several potentially relevant loci displaying a moderating effect of delivery mode on childhood intelligence (Table 2).

The top finding of this study is the interaction signal at rs17800861 in the intron of *GRIN2A* gene, identified in the models examining FSIQ (Table 2, Figure 1). *GRIN2A* encodes a subunit of the glutamate‐gated ion channel protein family member, *N*‐methyl‐d‐aspartate receptors (NMDARs), which plays a central role in the nervous system by regulating synaptic function (Cull‐Candy, Brickley, & Farrant, 2001). NMDARs are required for spatial learning and memory (Nakazawa, McHugh, Wilson, & Tonegawa, 2004) and are associated with fluid intelligence (problem‐solving ability; Kane & Engle, 2002). *GRIN2A* has also been linked to a form of benign epilepsy, co‐occurring with intellectual disability (Endele et al., 2010; Lesca et al., 2013; Reutlinger et al., 2010).

Our in silico examination of rs17800861 in HaploReg suggests that this SNP is located within active chromatin in the hippocampus, where neurogenesis might be affected by the mode of delivery (Seli & Horvath, 2013). Moreover, HaploReg also predicted that this SNP may alter the binding of NF‐kappaB factor that has been implicated in several cognition‐related phenotypes, including autism (Naik et al., 2011), schizophrenia (Song, Lv, Li, Hao, & Zhao, 2009), aggression (Brevik et al., 2016), and intellectual disability (Philippe et al., 2009). Interestingly, VD also correlates with increased activity of NF‐kappaB (Lee et al., 2003) and may modulate its expression (Cindrova‐Davies et al., 2007; Li & Karin, 1999).

Other notable interaction signals observed in this study are rs705670 on chromosome 9 for PIQ and rs1276529 on chromosome 6 for VIQ (Table 2). The first SNP (rs705670) is an intronic variant within a long non‐protein‐coding RNA (lncRNA) gene, *LINC01502*, while rs1276529 is an intergenic SNP located 141,817 bp to *RFPL4B* (Ret Finger Protein‐Like 4B) gene. lncRNAs have recently been implicated in a number of neuropsychiatric disorders where IQ is affected, such as schizophrenia, fragile X syndrome, and attention‐deficit/hyperactivity disorder (Ripke et al., 2014; Vondervoort et al., 2013; Wapinski & Chang, 2011; Zayats et al., 2015), while *RFPL4B* gene has been linked to language delay (Szafranski et al., 2015) and neuropsychiatric conditions where IQ is also affected (Hudson et al., 2014; Sun, Cheng, Zhang, & Xu, 2015).

Both mother and child experience certain physiological changes during VD that are absent during DCS (Kuguoglu, Yildiz, Tanir, & Demirbag, 2012), including the release of the hormone oxytocin that stimulates uterine muscular contractions (Carter, 2014). Oxytocin also protects the neonatal brain from delivery‐induced hypoxia, exerts an analgesic effect (Cavanagh et al., 2006; Mazzuca et al., 2011), and mediates an excitatory‐to‐inhibitory shift of GABAergic neurons following birth (Cavanagh et al., 2006; Cherubini, Gaiarsa, & Ben‐Ari, 1991). It has also been implicated in a number of neuropsychiatric disorders (Cochran, Fallon, Hill, & Frazier, 2013). The oxytocin receptor gene (*OXTR*) has been reported to be associated with IQ, cognition, and daily living skills (Lerer et al., 2008). Interestingly, NMDARs (encoded by the gene where one of our top hits is located) have been proposed to regulate synchronized activity of oxytocin neurons (Moos, Rossi, & Richard, 1997). Oxytocin injections to induce labor have previously been examined as a risk factor for neurodevelopmental disorders, revealing mixed results (Emberti Gialloreti et al., 2014; Glasson, 2013; Juul‐Dam, Townsend, & Courchesne, 2001; Oberg et al., 2016). Together, these studies highlight the need to directly examine a potential causal role of oxytocin on neurodevelopmental outcomes.

Other potential mediators between method of delivery and neurodevelopmental phenotypes include epigenetic changes, microbiome composition, and oxidative stress. Epigenetic changes play an important role in synaptic plasticity and memory formation (Grissom, Lubin, Gupta, & Parrish, 2011) as well as in a number of neuropsychiatric conditions where IQ is affected (Chaste & Leboyer, 2012; Hoffmann, Ziller, & Spengler, 2016; Siniscalco, Cirillo, Bradstreet, & Antonucci, 2013; Walton et al., 2016). Likewise, changes in methylation profiles have been linked to DCS (Schlinzig et al., 2009), though another study did not detect an effect between method of delivery and global methylation of DNA in the blood (Virani et al., 2012).

Since newborns are not exposed to the maternal gut flora during DCS, their microbiome may be different from that of babies delivered vaginally (Black et al., 2015; Salminen et al., 2004). Method of delivery may also affect gut colonization and immunological development of a child (Malamitsi‐Puchner et al., 2005; Vogl et al., 2006). Studies have shown that microbe content may influence behavior and affect brain function (Bercik et al., 2011; Smith, 2015; Yano et al., 2015) as well as the development of neurodevelopmental disorders where IQ is affected (Haavik, Halmøy, Hegvik, & Johansson, 2011).

Neonatal hypoxia may play a role in neurodevelopmental phenotypes, including deficits in memory and learning (Allin et al., 2004; Baoyuan, Salmaso, Komitova, Simonini, & Silbereis, 2011; Fagel et al., 2006; Ment et al., 2003). Our main hit has been linked to NF‐kappaB, whose expression has been reported to be altered by hypoxia during VD (Cindrova‐Davies et al., 2007; Li & Karin, 1999). Hypoxia‐related impairments may be reversed later in life, with recent findings suggesting that such recovery may be dependent on the expression of the genes affected by the method of delivery (Seli & Horvath, 2013). However, further studies are needed to explore whether this disturbance could have long‐lasting effect on the brain function.

The importance of VD has been highlighted in a recent large‐scale epidemiological study, where it was noted that children born by planned cesarean delivery had worse health outcomes than those born by VD, but not than those born by unscheduled cesarean section (Black et al., 2015).

Our study should be viewed in light of its limitations. As the genetics of intelligence are known to be polygenic (Plomin & Deary, 2014), our modest sample size limits our ability to detect interactions of expected small effect sizes. Thus, our results should be replicated and interpreted with caution. Nonetheless, our findings provide insight into the possible effect of method of delivery on the health outcomes in children.

Due to the small sample size and the lack of complete medical history, we could not distinguish between different types of cesarean sections (e.g., scheduled or emergency) that could potentially play an important role in the interpretation of our results as indicated by a recent large‐scale study (Black et al., 2015). The physiology of high‐risk babies delivered by planned cesarean section may also be considered as a confounding effect.

Further studies are needed to provide a conclusive answer to whether VD or DSC has fundamentally different impacts on the intelligence given individual genetic attributes. As the rates of DCSs continue to rise, the need for a better understanding of the physiology behind this method of delivery also continues to grow. Such studies would provide valuable insight for developing guidelines, informed decision‐making, and setting thresholds for the medical necessity of DCS.
