# Supplementary figures and images for: Moderating effect of mode of delivery on the genetics of intelligence: Explorative genome‐wide analyses in ALSPAC
Source: Brain Behav. 2018 Oct 31;8(12):e01144. doi: 10.1002/brb3.1144 (PMC6305932; doi:10.1002/brb3.1144)

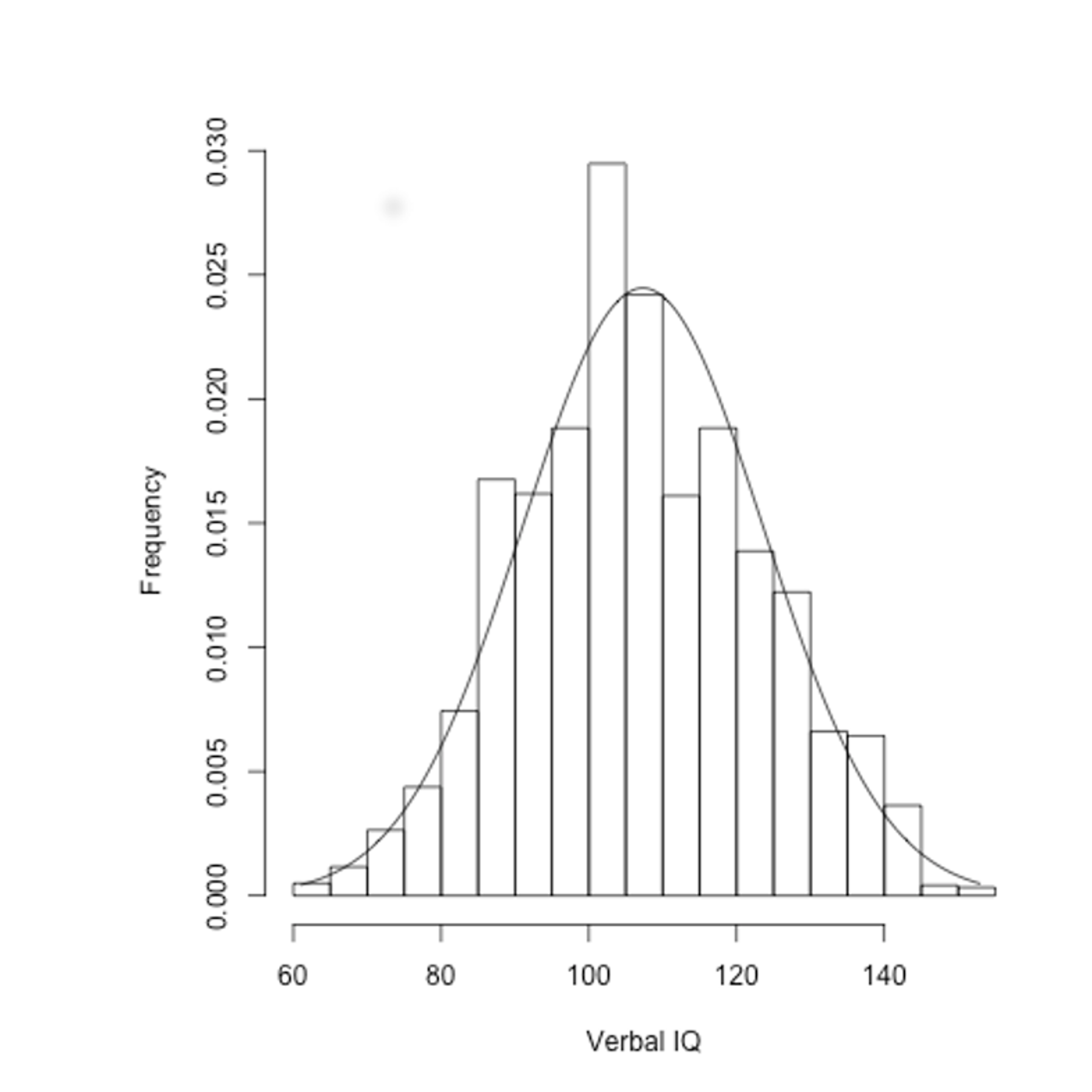

Supplement: Supplementary file 2 [file BRB3-8-e01144-s002.tiff]

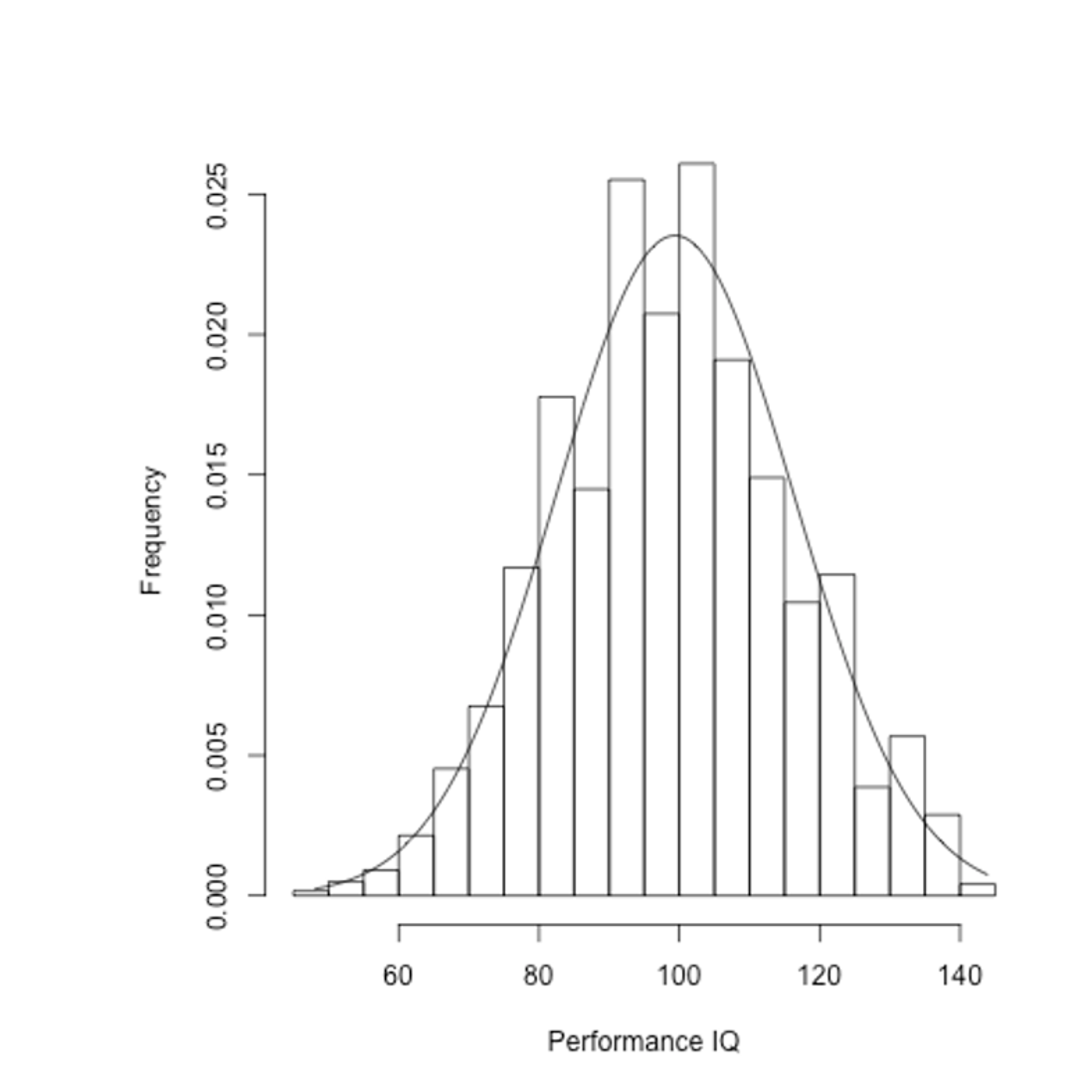

Supplement: Supplementary file 3 [file BRB3-8-e01144-s003.tiff]

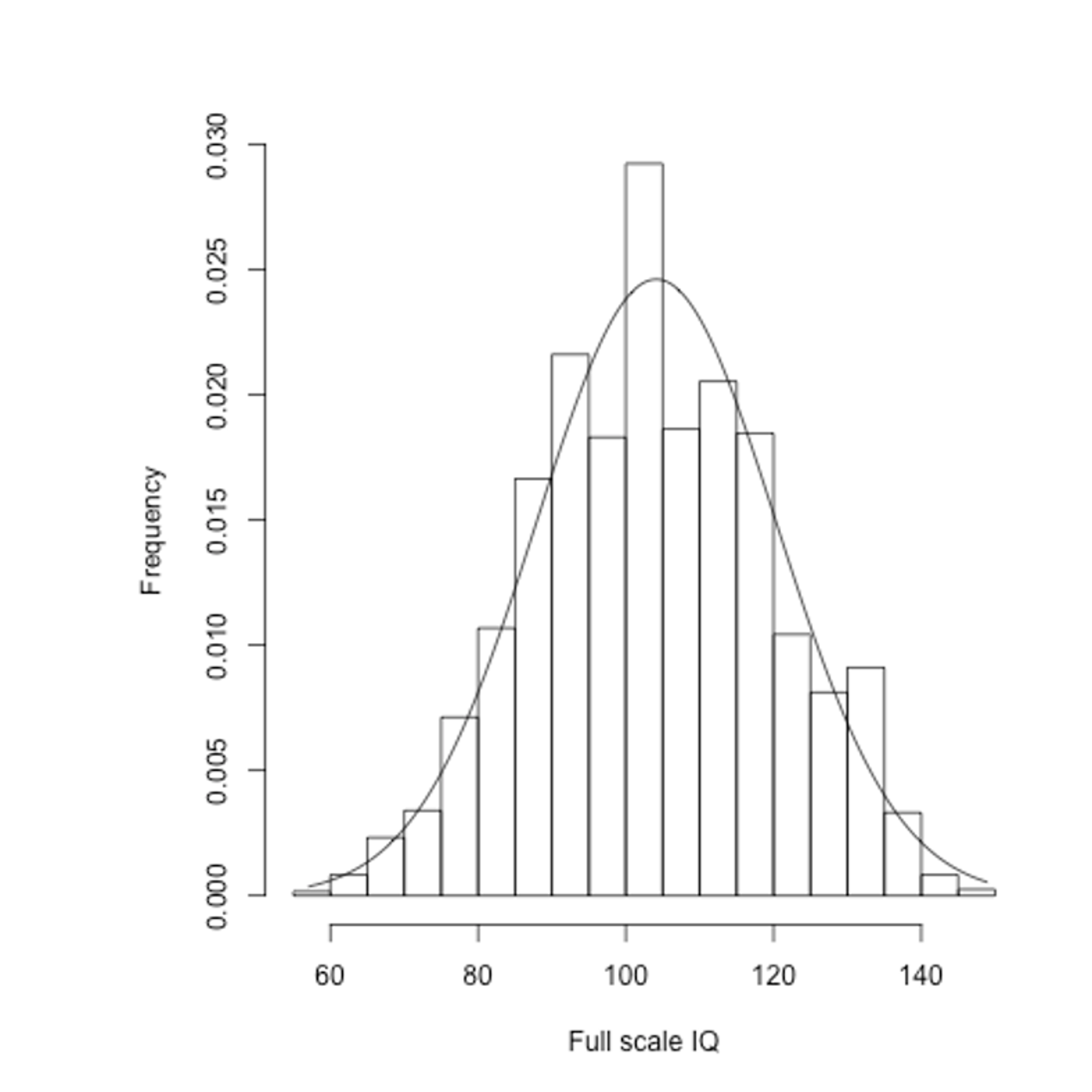

Supplement: Supplementary file 4 [file BRB3-8-e01144-s004.tiff]

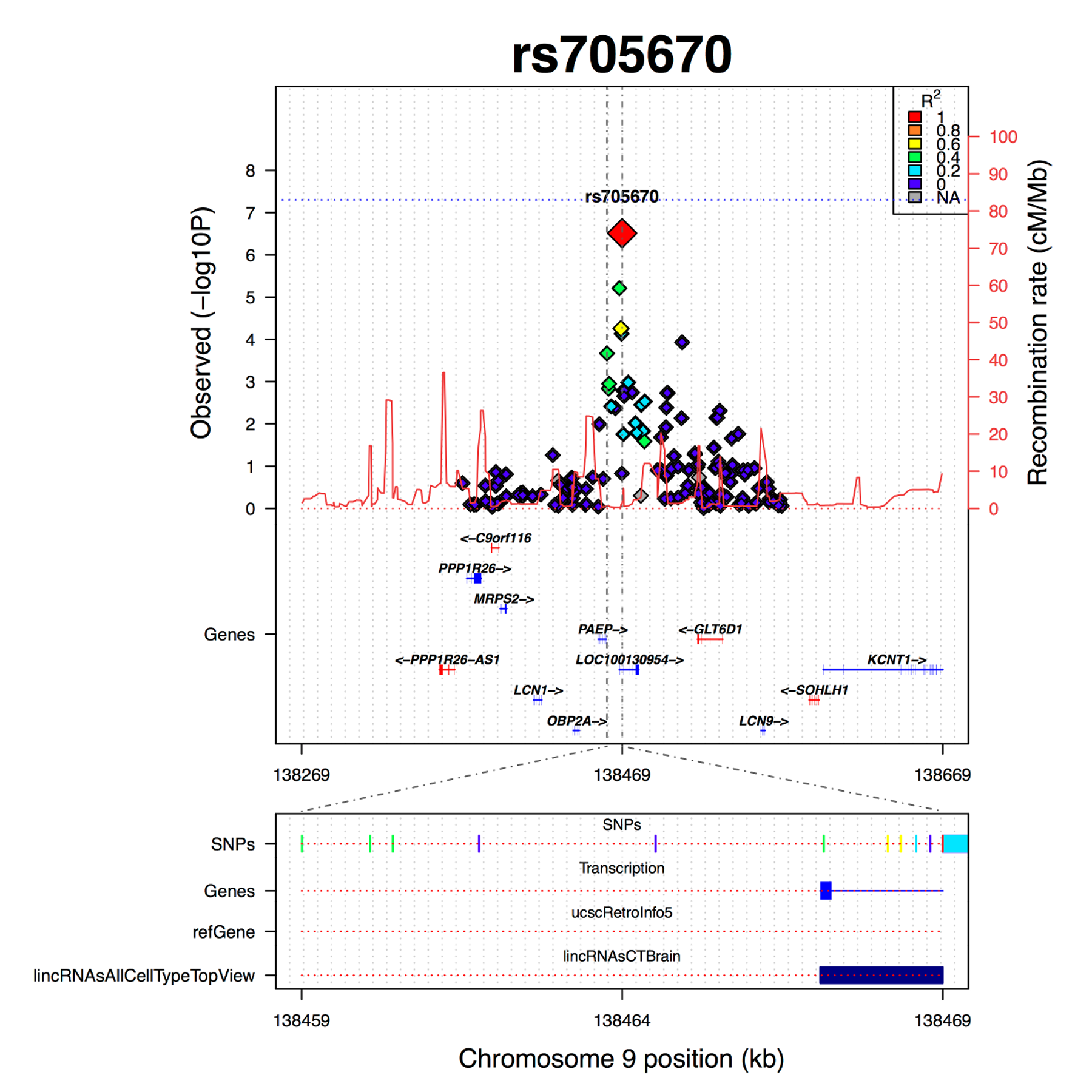

Supplement: Supplementary file 5 [file BRB3-8-e01144-s005.tiff]

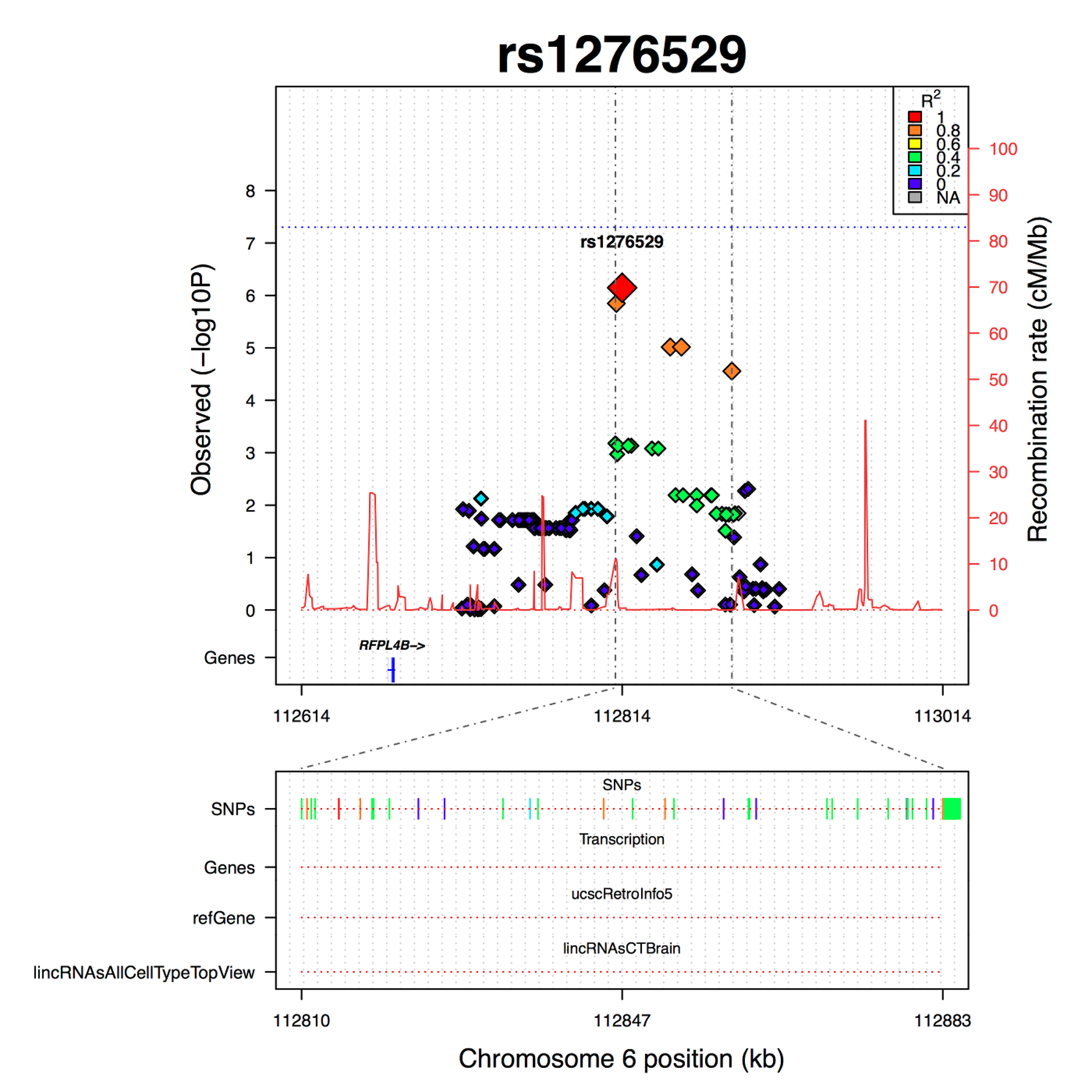

Supplement: Supplementary file 6 [file BRB3-8-e01144-s006.tiff]

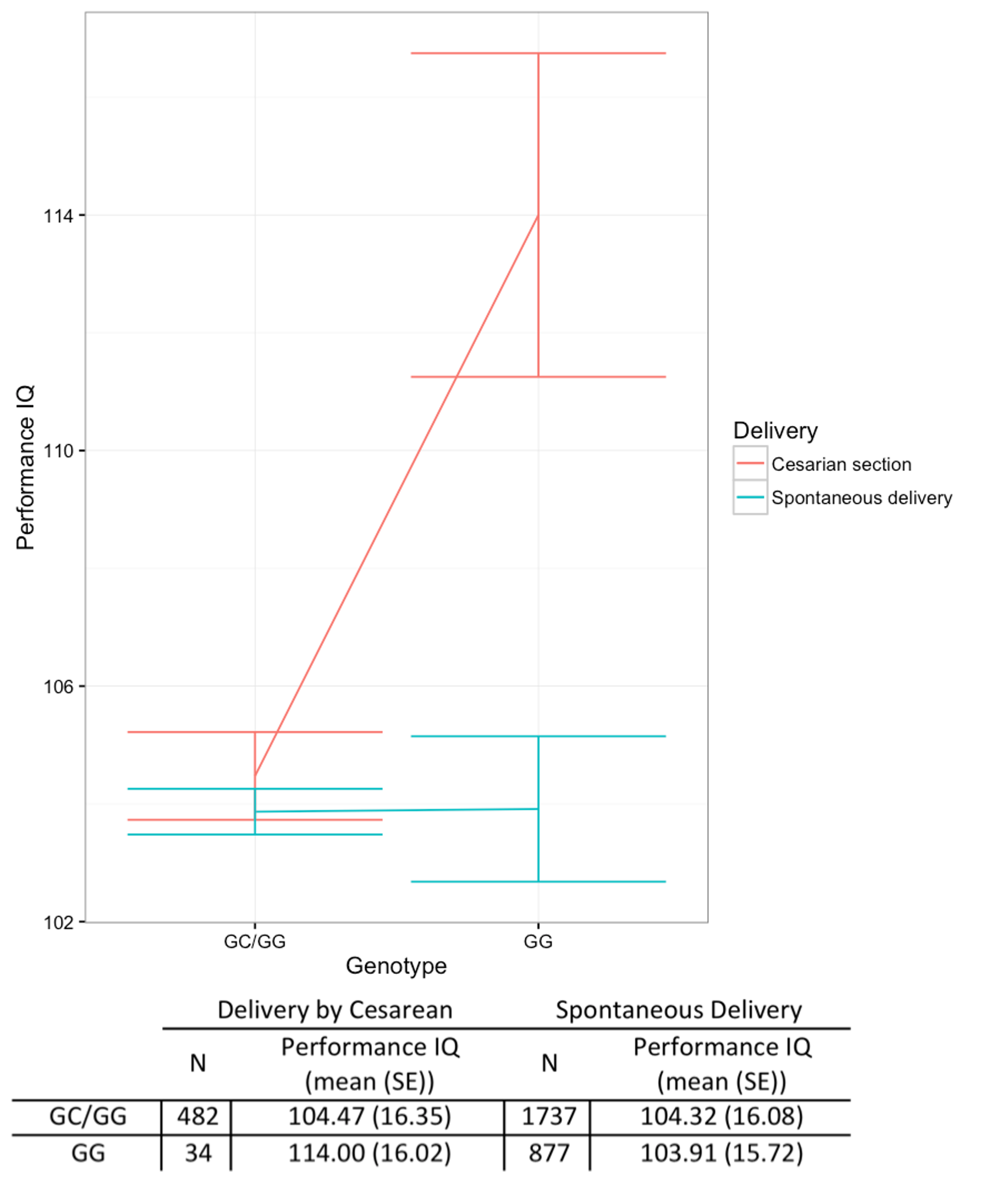

Supplement: Supplementary file 7 [file BRB3-8-e01144-s007.tiff]

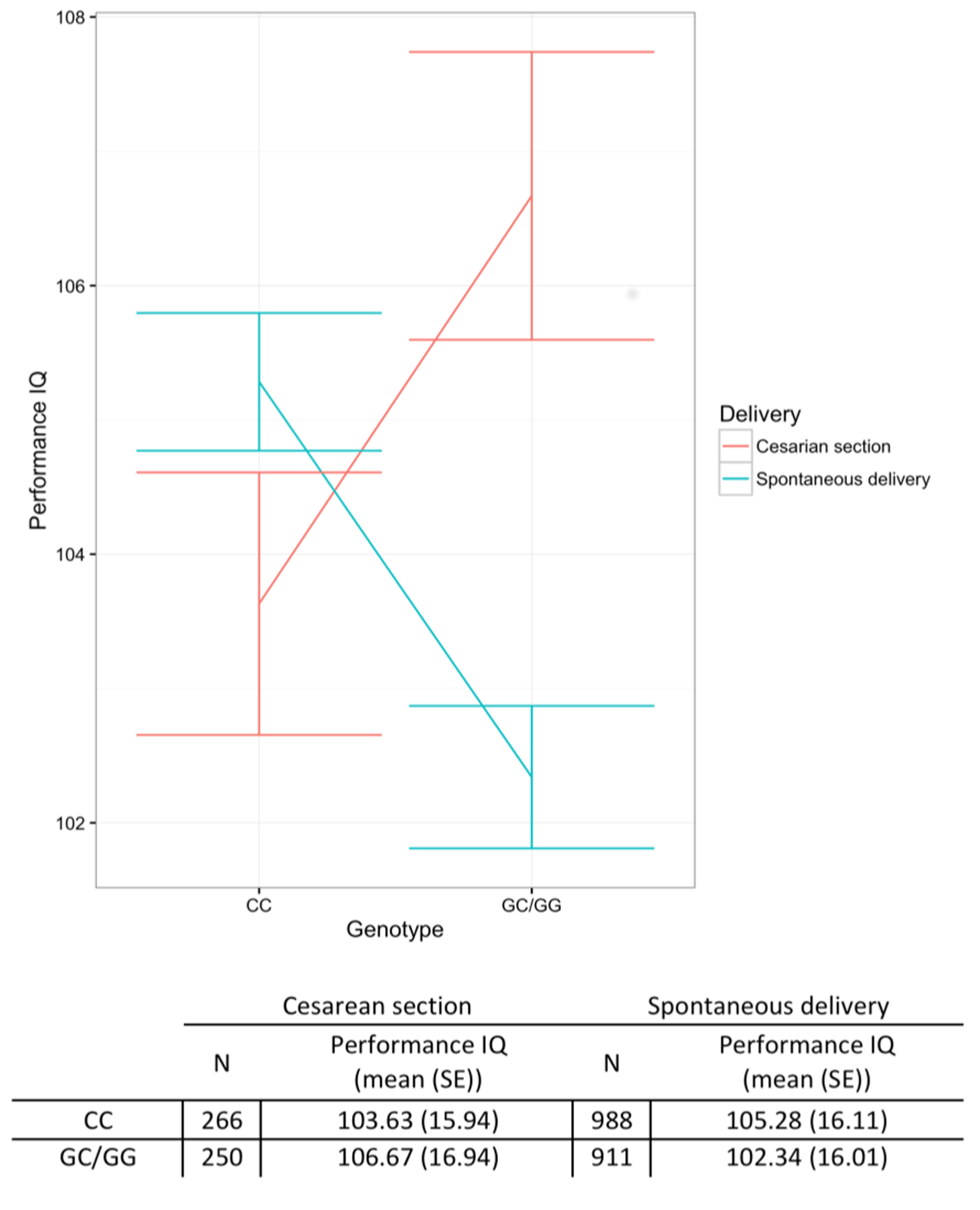

Supplement: Supplementary file 8 [file BRB3-8-e01144-s008.tiff]

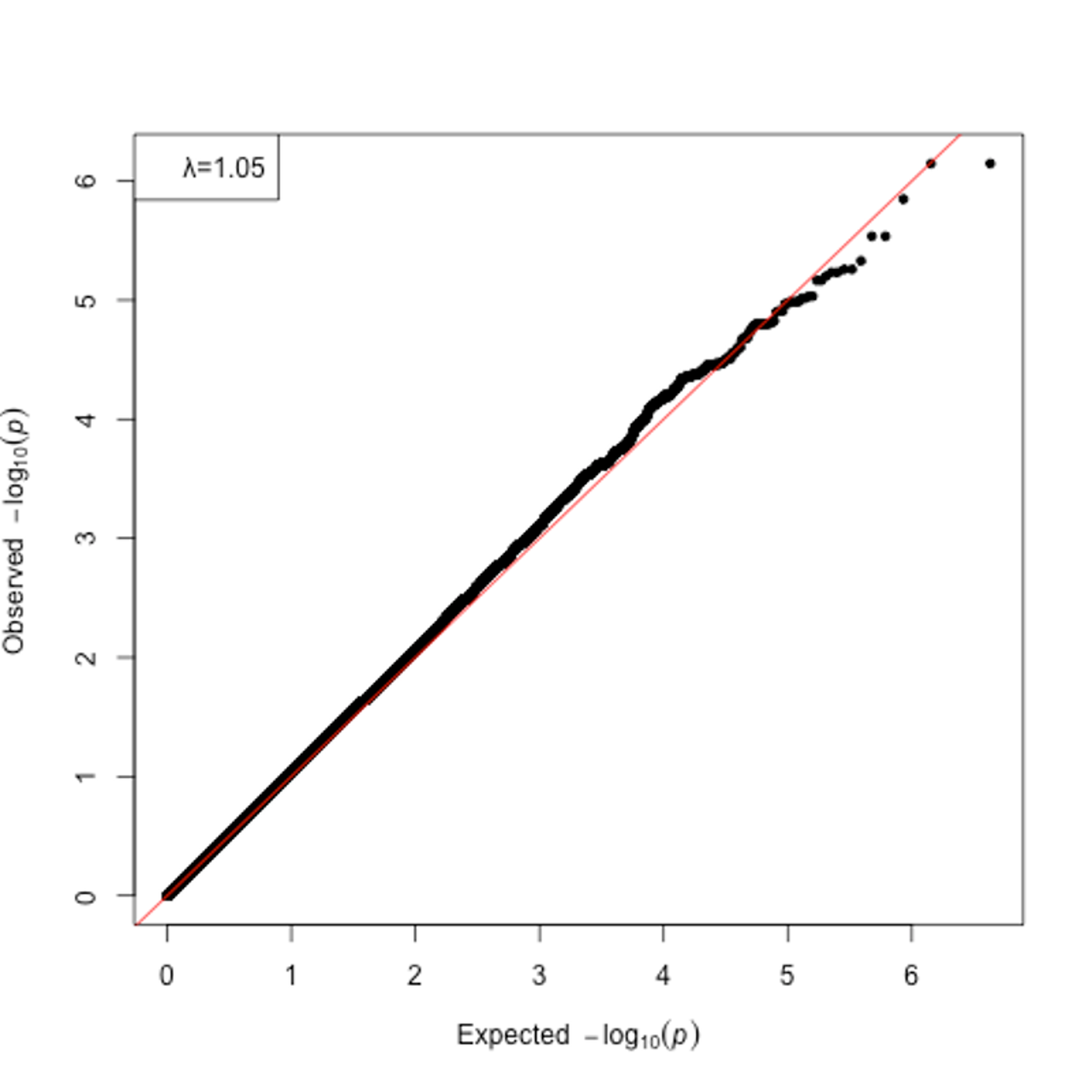

Supplement: Supplementary file 9 [file BRB3-8-e01144-s009.tiff]

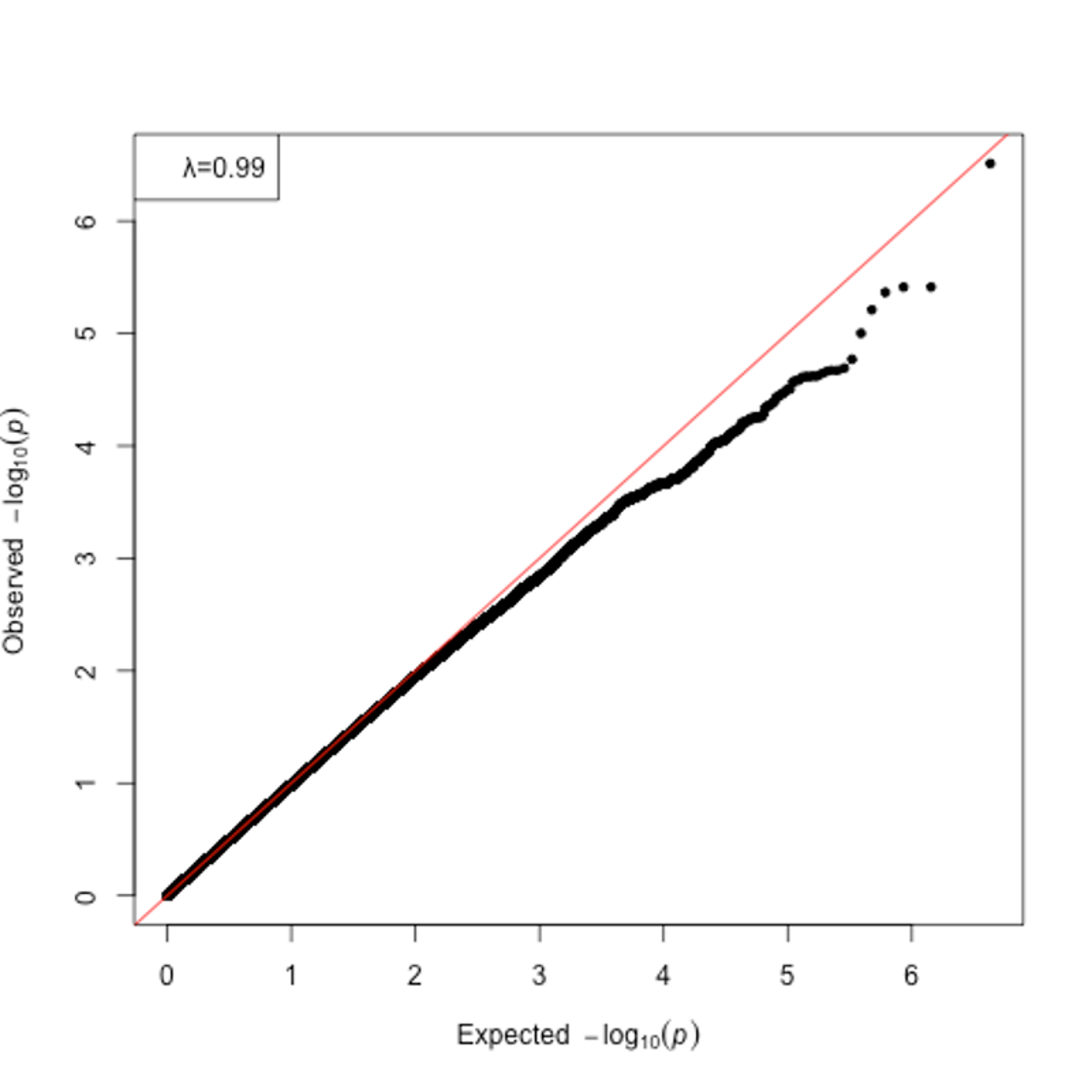

Supplement: Supplementary file 10 [file BRB3-8-e01144-s010.tiff]

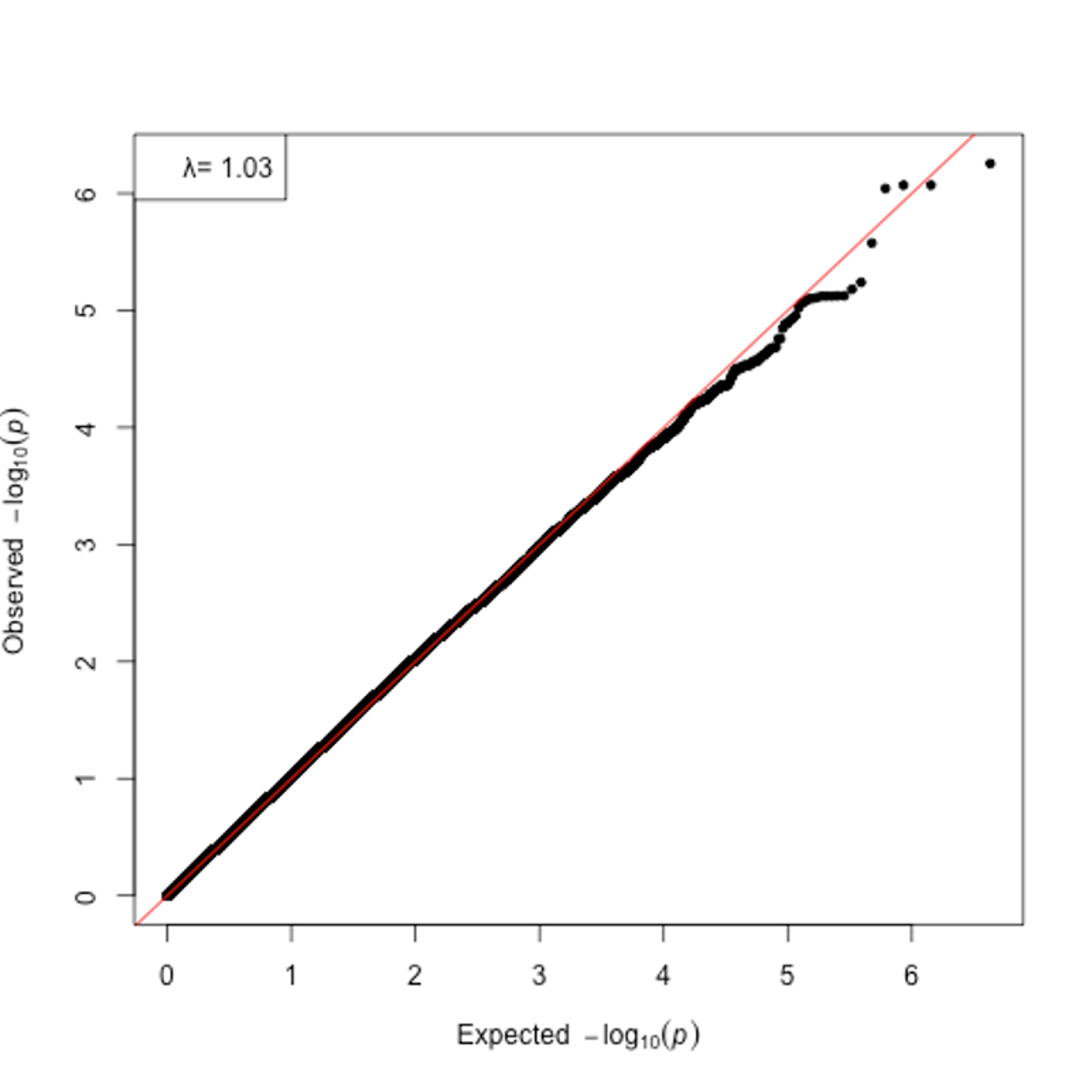

Supplement: Supplementary file 11 [file BRB3-8-e01144-s011.tiff]

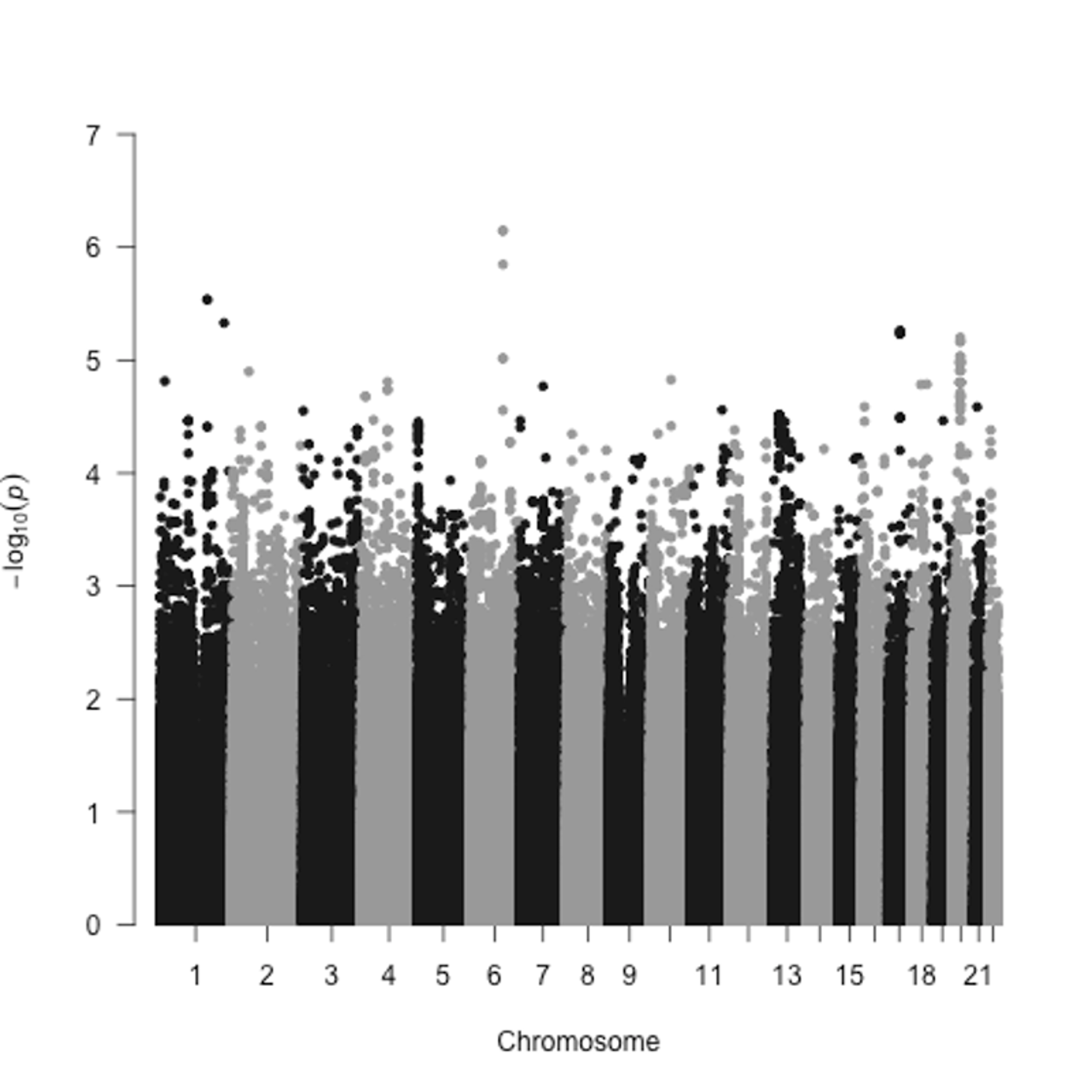

Supplement: Supplementary file 12 [file BRB3-8-e01144-s012.tiff]

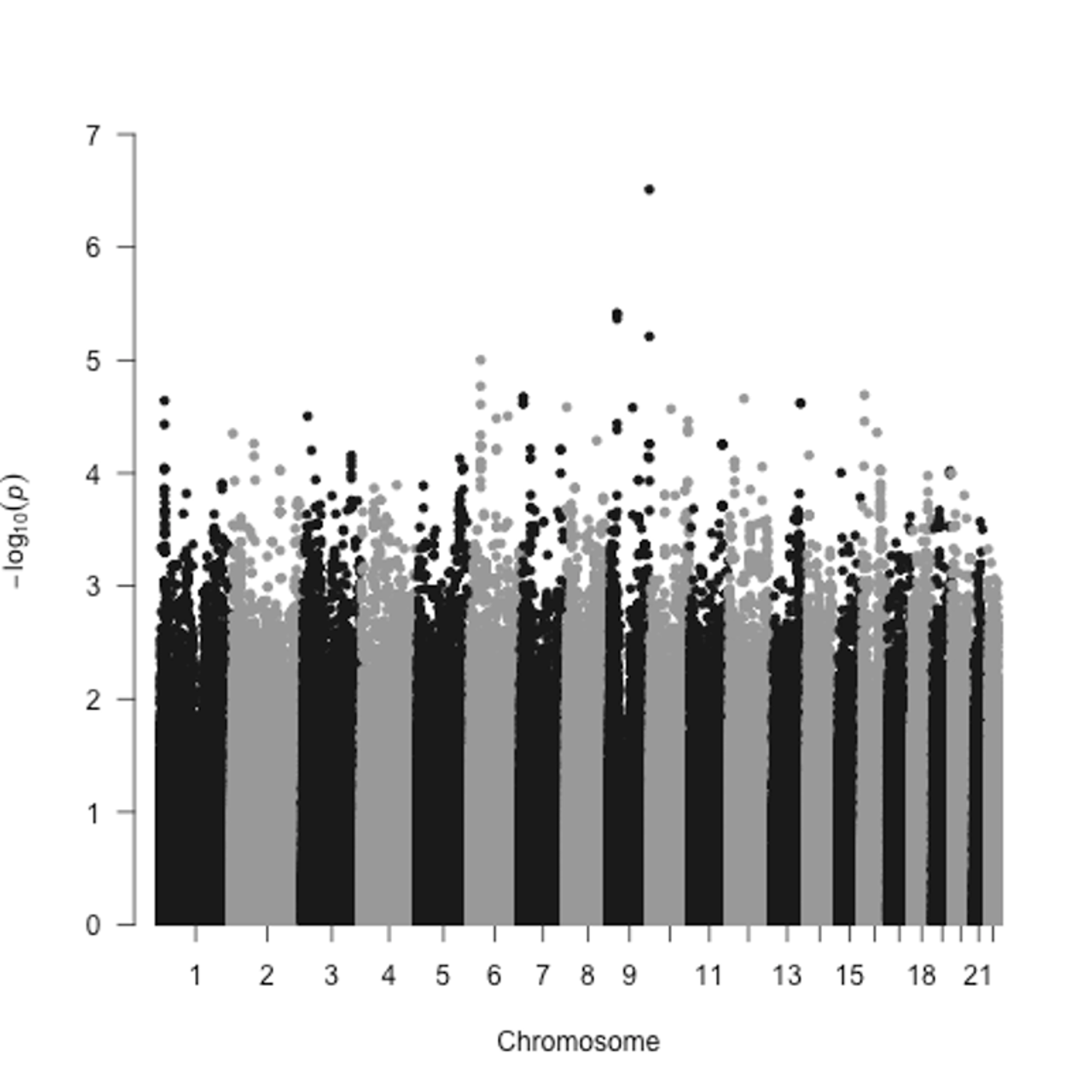

Supplement: Supplementary file 13 [file BRB3-8-e01144-s013.tiff]

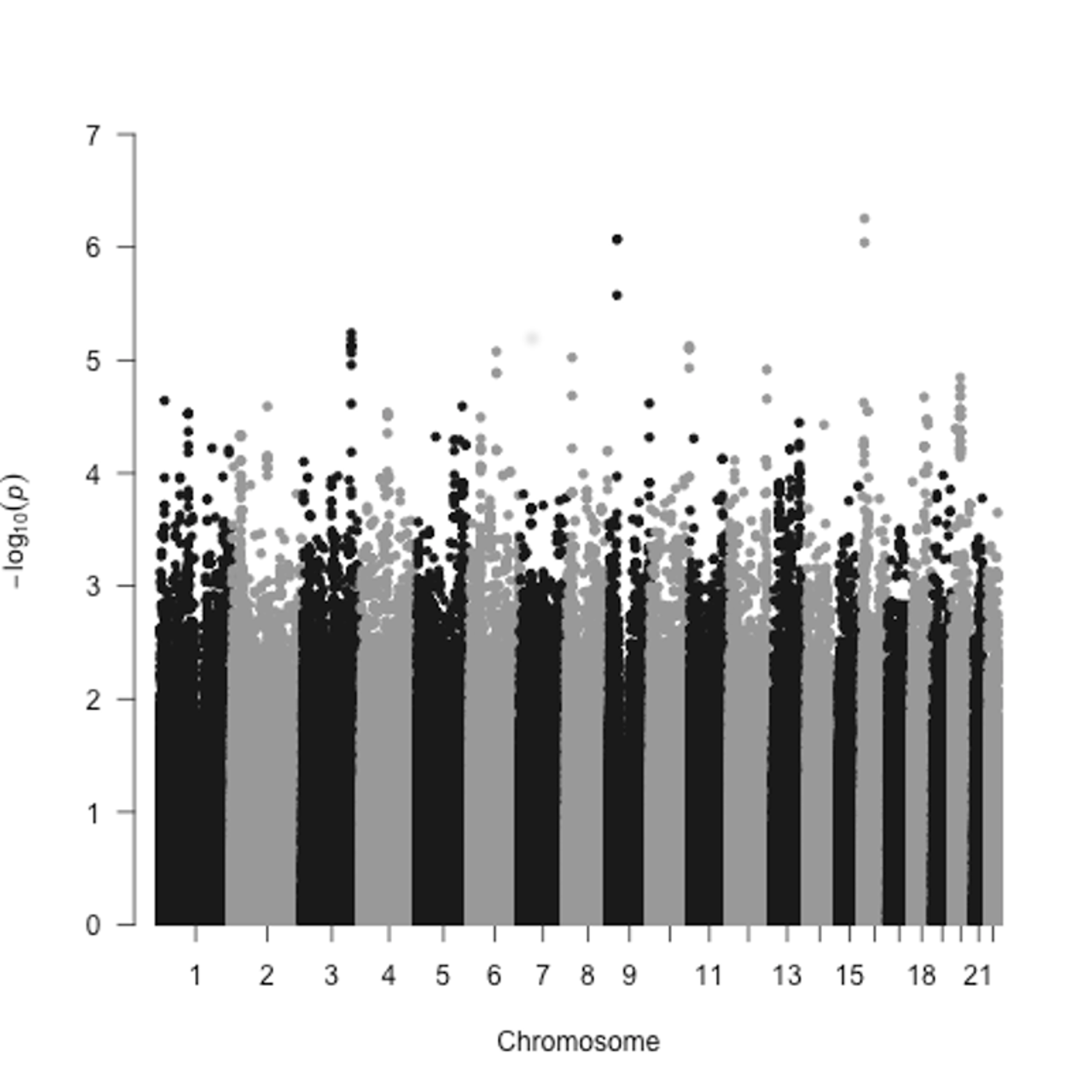

Supplement: Supplementary file 14 [file BRB3-8-e01144-s014.tiff]
