# Supplementary material for: Moderating effect of mode of delivery on the genetics of intelligence: Explorative genome‐wide analyses in ALSPAC
Source: Brain Behav. 2018 Oct 31;8(12):e01144. doi: 10.1002/brb3.1144 (PMC6305932; doi:10.1002/brb3.1144)
Supplement: Supplementary file 15 [file BRB3-8-e01144-s015.docx]

**S1 Table. Correlation between perinatal factors examined in this study.**

|  | Birth weight | Birth length | Apgar at 1 minute | Delivery by caesarean section | Gestational age |
| --- | --- | --- | --- | --- | --- |
| Birth weight | 1 | 0.81 | 0.09 | 0.04 | 0.41 |
|  |  |  |  |  |  |
| Birth length | 0.81 | 1 | 0.04 | 0.05 | 0.45 |
|  |  |  |  |  |  |
| Apgar at 1 minute | 0.09 | 0.04 | 1 | 0.22 | 0.05 |
|  |  |  |  |  |  |
| Delivery by caesarean section | 0.04 | 0.05 | 0.22 | 1 | 0.11 |
|  |  |  |  |  |  |
| Gestational age | 0.41 | 0.45 | 0.05 | 0.11 | 1 |
|  |  |  |  |  |  |

Spearman´s test was used for continuous variables and Pearson test was used for categorical variables.
