# Supplementary material for: Moderating effect of mode of delivery on the genetics of intelligence: Explorative genome‐wide analyses in ALSPAC
Source: Brain Behav. 2018 Oct 31;8(12):e01144. doi: 10.1002/brb3.1144 (PMC6305932; doi:10.1002/brb3.1144)
Supplement: Supplementary file 16 [file BRB3-8-e01144-s016.docx]

**S2 Table. Top interaction hits (p<1.00E-04) observed for the VIQ scores.**

| Verbal IQ | | | | | | | | | |
| --- | --- | --- | --- | --- | --- | --- | --- | --- | --- |
|  | | | | Crude model | | | Adjusted model | | |
| SNP | CHR | BP | Effect allele | β | C.I. | P value | β | C.I. | P value |
| rs1276529 | 6 | 112921192 | G | -2.04 | -2.86 - -1.22 | 1.10E-06 | -2.09 | -2.91 - -1.26 | 7.13E-07 |
| rs1706066 | 6 | 112923618 | G | -2.04 | -2.86 - -1.22 | 1.10E-06 | -2.09 | -2.91 - -1.26 | 7.13E-07 |
| rs1276583 | 6 | 112917585 | G | -1.98 | -2.79 - -1.18 | 1.33E-06 | -1.99 | -2.79 - -1.18 | 1.42E-06 |
| rs1506944 | 1 | 163484162 | C | -1.73 | -2.45 - -1.00 | 3.64E-06 | -1.75 | -2.48 - -1.02 | 2.91E-06 |
| rs1506946 | 1 | 163483980 | A | -1.73 | -2.45 - -1.00 | 3.64E-06 | -1.75 | -2.48 - -1.02 | 2.91E-06 |
| rs17464857 | 1 | 220829332 | G | 2.82 | 1.60 - 4.05 | 6.38E-06 | 2.87 | 1.64 - 4.10 | 4.68E-06 |
| rs4968308 | 17 | 42633006 | T | -1.79 | -2.58 - -0.99 | 1.09E-05 | -1.85 | -2.64 - -1.05 | 5.51E-06 |
| rs858671 | 17 | 42626679 | A | -1.79 | -2.58 - -0.99 | 1.09E-05 | -1.85 | -2.64 - -1.05 | 5.51E-06 |
| rs11079763 | 17 | 42643788 | T | -1.78 | -2.57 - -0.99 | 1.15E-05 | -1.84 | -2.64 - -1.05 | 5.85E-06 |
| rs9894365 | 17 | 42639786 | G | -1.78 | -2.57 - -0.99 | 1.15E-05 | -1.84 | -2.64 - -1.05 | 5.85E-06 |
| rs6060973 | 20 | 29875483 | T | -3.33 | -4.75 - -1.90 | 5.16E-06 | -3.32 | -4.75 - -1.88 | 6.28E-06 |
| rs6058471 | 20 | 29883845 | G | -3.13 | -4.50 - -1.76 | 8.17E-06 | -3.17 | -4.55 - -1.79 | 6.83E-06 |
| rs6060980 | 20 | 29883615 | G | -3.13 | -4.50 - -1.76 | 8.17E-06 | -3.17 | -4.55 - -1.79 | 6.83E-06 |
| rs11905334 | 20 | 29888704 | G | -3.07 | -4.44 - -1.71 | 1.10E-05 | -3.11 | -4.49 - -1.74 | 9.27E-06 |
| rs7269370 | 20 | 29886172 | A | -3.07 | -4.44 - -1.71 | 1.10E-05 | -3.11 | -4.49 - -1.74 | 9.27E-06 |
| rs1276541 | 6 | 112951194 | C | -1.81 | -2.63 - -1.00 | 1.40E-05 | -1.86 | -2.67 - -1.04 | 9.64E-06 |
| rs1706043 | 6 | 112958155 | G | -1.81 | -2.63 - -1.00 | 1.40E-05 | -1.86 | -2.67 - -1.04 | 9.64E-06 |
| rs12479946 | 20 | 29748070 | C | -2.98 | -4.32 - -1.65 | 1.21E-05 | -3.02 | -4.36 - -1.68 | 1.03E-05 |
| rs12480630 | 20 | 29747273 | T | -2.98 | -4.32 - -1.65 | 1.21E-05 | -3.02 | -4.36 - -1.68 | 1.03E-05 |
| rs17253922 | 20 | 29751357 | C | -2.98 | -4.32 - -1.65 | 1.21E-05 | -3.02 | -4.36 - -1.68 | 1.03E-05 |
| rs6060855 | 20 | 29762606 | T | -2.98 | -4.32 - -1.65 | 1.21E-05 | -3.02 | -4.36 - -1.68 | 1.03E-05 |
| rs6058316 | 20 | 29719634 | C | -2.98 | -4.31 - -1.64 | 1.26E-05 | -3.02 | -4.36 - -1.68 | 1.07E-05 |
| rs6058381 | 20 | 29739567 | G | -2.98 | -4.31 - -1.64 | 1.26E-05 | -3.02 | -4.36 - -1.68 | 1.07E-05 |
| rs6060477 | 20 | 29700406 | G | -2.96 | -4.29 - -1.62 | 1.44E-05 | -2.99 | -4.33 - -1.65 | 1.24E-05 |
| rs6060489 | 20 | 29701566 | A | -2.96 | -4.29 - -1.62 | 1.44E-05 | -2.99 | -4.33 - -1.65 | 1.24E-05 |
| rs6060531 | 20 | 29707019 | A | -2.96 | -4.29 - -1.62 | 1.44E-05 | -2.99 | -4.33 - -1.65 | 1.24E-05 |
| rs17049508 | 2 | 58538443 | G | 1.89 | 1.05 - 2.74 | 1.28E-05 | 1.90 | 1.05 - 2.75 | 1.26E-05 |
| rs10823448 | 10 | 71378411 | G | -2.50 | -3.60 - -1.39 | 1.02E-05 | -2.46 | -3.56 - -1.35 | 1.49E-05 |
| rs10907288 | 1 | 18371909 | C | -2.08 | -2.99 - -1.18 | 7.09E-06 | -2.02 | -2.93 - -1.11 | 1.53E-05 |
| rs1367789 | 4 | 90428182 | C | -2.14 | -3.15 - -1.13 | 3.17E-05 | -2.23 | -3.24 - -1.22 | 1.55E-05 |
| rs11167292 | 20 | 29807763 | C | -2.93 | -4.27 - -1.59 | 1.85E-05 | -2.97 | -4.31 - -1.62 | 1.58E-05 |
| rs11907631 | 20 | 29822157 | C | -2.93 | -4.27 - -1.59 | 1.85E-05 | -2.97 | -4.31 - -1.62 | 1.58E-05 |
| rs12481724 | 20 | 29807408 | G | -2.93 | -4.27 - -1.59 | 1.85E-05 | -2.97 | -4.31 - -1.62 | 1.58E-05 |
| rs17339879 | 20 | 29834553 | T | -2.93 | -4.27 - -1.59 | 1.85E-05 | -2.97 | -4.31 - -1.62 | 1.58E-05 |
| rs2182967 | 20 | 29803046 | G | -2.93 | -4.27 - -1.59 | 1.85E-05 | -2.97 | -4.31 - -1.62 | 1.58E-05 |
| rs6058461 | 20 | 29827905 | G | -2.93 | -4.27 - -1.59 | 1.85E-05 | -2.97 | -4.31 - -1.62 | 1.58E-05 |
| rs6060913 | 20 | 29790501 | C | -2.93 | -4.27 - -1.59 | 1.85E-05 | -2.97 | -4.31 - -1.62 | 1.58E-05 |
| rs6060943 | 20 | 29835115 | C | -2.93 | -4.27 - -1.59 | 1.85E-05 | -2.97 | -4.31 - -1.62 | 1.58E-05 |
| rs7270207 | 20 | 29784544 | C | -2.93 | -4.27 - -1.59 | 1.85E-05 | -2.97 | -4.31 - -1.62 | 1.58E-05 |
| rs9951126 | 18 | 55635276 | T | -1.69 | -2.45 - -0.93 | 1.47E-05 | -1.69 | -2.46 - -0.92 | 1.63E-05 |
| rs4800089 | 18 | 35132932 | T | 1.71 | 0.89 - 2.53 | 4.21E-05 | 1.81 | 0.99 - 2.63 | 1.64E-05 |
| rs1403880 | 7 | 79185392 | G | -2.65 | -3.91 - -1.39 | 3.96E-05 | -2.79 | -4.05 - -1.52 | 1.70E-05 |
| rs1019875 | 4 | 90433131 | T | -2.12 | -3.13 - -1.12 | 3.71E-05 | -2.21 | -3.23 - -1.20 | 1.83E-05 |
| rs1431546 | 4 | 90432789 | G | -2.12 | -3.13 - -1.12 | 3.71E-05 | -2.21 | -3.23 - -1.20 | 1.83E-05 |
| rs729838 | 20 | 29727189 | A | -2.81 | -4.10 - -1.51 | 2.25E-05 | -2.84 | -4.14 - -1.54 | 1.95E-05 |
| rs6060963 | 20 | 29862025 | C | -2.89 | -4.23 - -1.55 | 2.42E-05 | -2.93 | -4.27 - -1.58 | 2.09E-05 |
| rs6060964 | 20 | 29864318 | A | -2.89 | -4.23 - -1.55 | 2.42E-05 | -2.93 | -4.27 - -1.58 | 2.09E-05 |
| rs6060965 | 20 | 29869759 | G | -2.89 | -4.23 - -1.55 | 2.42E-05 | -2.93 | -4.27 - -1.58 | 2.09E-05 |
| rs4698325 | 4 | 14831535 | A | -2.23 | -3.23 - -1.23 | 1.18E-05 | -2.18 | -3.18 - -1.18 | 2.10E-05 |
| rs6449117 | 4 | 14832737 | G | -2.23 | -3.23 - -1.23 | 1.18E-05 | -2.18 | -3.18 - -1.18 | 2.10E-05 |
| rs6060951 | 20 | 29842712 | T | -2.88 | -4.22 - -1.54 | 2.56E-05 | -2.92 | -4.27 - -1.58 | 2.20E-05 |
| rs11907253 | 20 | 29696334 | A | -2.85 | -4.18 - -1.52 | 2.81E-05 | -2.88 | -4.22 - -1.55 | 2.49E-05 |
| rs1484998 | 20 | 29695950 | C | -2.85 | -4.18 - -1.52 | 2.81E-05 | -2.88 | -4.22 - -1.55 | 2.49E-05 |
| rs6060454 | 20 | 29696565 | G | -2.85 | -4.18 - -1.52 | 2.81E-05 | -2.88 | -4.22 - -1.55 | 2.49E-05 |
| rs17800861 | 16 | 9861173 | A | -2.67 | -3.92 - -1.42 | 3.15E-05 | -2.70 | -3.96 - -1.45 | 2.59E-05 |
| rs2829350 | 21 | 25065115 | C | -1.63 | -2.38 - -0.87 | 2.43E-05 | -1.63 | -2.38 - -0.87 | 2.60E-05 |
| rs6060972 | 20 | 29875261 | T | -2.93 | -4.28 - -1.58 | 2.21E-05 | -2.91 | -4.27 - -1.56 | 2.71E-05 |
| rs12364447 | 11 | 110445987 | A | -2.39 | -3.53 - -1.26 | 3.75E-05 | -2.43 | -3.57 - -1.30 | 2.76E-05 |
| rs1276535 | 6 | 112989635 | A | -1.75 | -2.59 - -0.92 | 3.91E-05 | -1.80 | -2.63 - -0.96 | 2.78E-05 |
| rs6060911 | 20 | 29787599 | C | -2.87 | -4.22 - -1.52 | 3.20E-05 | -2.91 | -4.27 - -1.55 | 2.78E-05 |
| rs9814431 | 3 | 1963471 | C | -2.71 | -3.96 - -1.45 | 2.46E-05 | -2.69 | -3.94 - -1.43 | 2.81E-05 |
| rs7334651 | 13 | 39197252 | C | -1.59 | -2.35 - -0.83 | 4.39E-05 | -1.63 | -2.39 - -0.86 | 3.08E-05 |
| rs7998641 | 13 | 39225895 | T | -1.59 | -2.35 - -0.83 | 4.39E-05 | -1.63 | -2.39 - -0.86 | 3.08E-05 |
| rs9548916 | 13 | 39220095 | C | -1.59 | -2.35 - -0.83 | 4.39E-05 | -1.63 | -2.39 - -0.86 | 3.08E-05 |
| rs9548917 | 13 | 39221238 | T | -1.59 | -2.35 - -0.83 | 4.39E-05 | -1.63 | -2.39 - -0.86 | 3.08E-05 |
| rs9548920 | 13 | 39225460 | G | -1.59 | -2.35 - -0.83 | 4.39E-05 | -1.63 | -2.39 - -0.86 | 3.08E-05 |
| rs9548926 | 13 | 39231767 | C | -1.59 | -2.35 - -0.83 | 4.39E-05 | -1.63 | -2.39 - -0.86 | 3.08E-05 |
| rs16941650 | 17 | 42633789 | A | -1.79 | -2.66 - -0.93 | 5.11E-05 | -1.84 | -2.71 - -0.98 | 3.21E-05 |
| rs858670 | 17 | 42626763 | A | -1.79 | -2.66 - -0.93 | 5.11E-05 | -1.84 | -2.71 - -0.98 | 3.21E-05 |
| rs1106950 | 17 | 42636404 | A | -1.79 | -2.66 - -0.93 | 5.18E-05 | -1.84 | -2.71 - -0.98 | 3.25E-05 |
| rs6058274 | 20 | 29702707 | T | -2.79 | -4.10 - -1.49 | 2.70E-05 | -2.78 | -4.09 - -1.47 | 3.32E-05 |
| rs10023065 | 4 | 42564694 | T | 1.58 | 0.83 - 2.34 | 4.38E-05 | 1.61 | 0.85 - 2.37 | 3.39E-05 |
| rs10952167 | 7 | 1560184 | A | 1.70 | 0.91 - 2.50 | 2.53E-05 | 1.68 | 0.89 - 2.47 | 3.41E-05 |
| rs12480322 | 20 | 29900302 | C | -2.86 | -4.21 - -1.50 | 3.69E-05 | -2.88 | -4.24 - -1.52 | 3.41E-05 |
| rs12480325 | 20 | 29900327 | A | -2.86 | -4.21 - -1.50 | 3.69E-05 | -2.88 | -4.24 - -1.52 | 3.41E-05 |
| rs162825 | 19 | 35076443 | C | 2.38 | 1.25 - 3.50 | 3.51E-05 | 2.39 | 1.26 - 3.52 | 3.42E-05 |
| rs17381294 | 1 | 98376789 | A | -1.80 | -2.66 - -0.95 | 3.68E-05 | -1.81 | -2.67 - -0.96 | 3.44E-05 |
| rs17381509 | 1 | 98395255 | G | -1.80 | -2.65 - -0.94 | 3.92E-05 | -1.81 | -2.67 - -0.96 | 3.44E-05 |
| rs17381657 | 1 | 98417071 | G | -1.80 | -2.65 - -0.94 | 3.92E-05 | -1.81 | -2.67 - -0.96 | 3.44E-05 |
| rs963852 | 1 | 98401865 | G | -1.80 | -2.65 - -0.94 | 3.92E-05 | -1.81 | -2.67 - -0.96 | 3.44E-05 |
| rs4312180 | 13 | 39138381 | C | -1.57 | -2.33 - -0.81 | 4.87E-05 | -1.61 | -2.37 - -0.85 | 3.45E-05 |
| rs9532414 | 13 | 39134995 | T | -1.57 | -2.33 - -0.81 | 4.87E-05 | -1.61 | -2.37 - -0.85 | 3.45E-05 |
| rs9548875 | 13 | 39143287 | T | -1.57 | -2.33 - -0.81 | 4.87E-05 | -1.61 | -2.37 - -0.85 | 3.45E-05 |
| rs8058978 | 16 | 9857437 | G | -2.62 | -3.86 - -1.37 | 4.24E-05 | -2.65 | -3.90 - -1.40 | 3.48E-05 |
| rs2138856 | 5 | 4021962 | A | 1.63 | 0.85 - 2.42 | 4.35E-05 | 1.66 | 0.88 - 2.45 | 3.51E-05 |
| rs4374050 | 13 | 58974986 | G | -1.71 | -2.52 - -0.89 | 4.64E-05 | -1.73 | -2.56 - -0.91 | 3.52E-05 |
| rs7327779 | 13 | 39127724 | G | -1.57 | -2.33 - -0.81 | 5.04E-05 | -1.61 | -2.36 - -0.85 | 3.53E-05 |
| rs9548866 | 13 | 39127420 | T | -1.57 | -2.33 - -0.81 | 5.04E-05 | -1.61 | -2.36 - -0.85 | 3.53E-05 |
| rs9548867 | 13 | 39127568 | T | -1.57 | -2.33 - -0.81 | 5.04E-05 | -1.61 | -2.36 - -0.85 | 3.53E-05 |
| rs9548868 | 13 | 39127585 | T | -1.57 | -2.33 - -0.81 | 5.04E-05 | -1.61 | -2.36 - -0.85 | 3.53E-05 |
| rs3812883 | 13 | 39127957 | A | -1.57 | -2.33 - -0.81 | 5.05E-05 | -1.60 | -2.36 - -0.85 | 3.54E-05 |
| rs3812884 | 13 | 39128043 | T | -1.57 | -2.33 - -0.81 | 5.05E-05 | -1.60 | -2.36 - -0.85 | 3.54E-05 |
| rs3812885 | 13 | 39128122 | C | -1.57 | -2.33 - -0.81 | 5.05E-05 | -1.60 | -2.36 - -0.85 | 3.54E-05 |
| rs3812886 | 13 | 39128216 | A | -1.57 | -2.33 - -0.81 | 5.05E-05 | -1.60 | -2.36 - -0.85 | 3.54E-05 |
| rs3812887 | 13 | 39128229 | T | -1.57 | -2.33 - -0.81 | 5.05E-05 | -1.60 | -2.36 - -0.85 | 3.54E-05 |
| rs3812888 | 13 | 39128294 | C | -1.57 | -2.33 - -0.81 | 5.05E-05 | -1.60 | -2.36 - -0.85 | 3.54E-05 |
| rs9548869 | 13 | 39128705 | A | -1.57 | -2.33 - -0.81 | 5.05E-05 | -1.60 | -2.36 - -0.85 | 3.54E-05 |
| rs2932330 | 5 | 3984045 | A | 1.67 | 0.87 - 2.46 | 4.05E-05 | 1.68 | 0.88 - 2.47 | 3.76E-05 |
| rs505800 | 5 | 3980833 | G | 1.67 | 0.87 - 2.46 | 4.05E-05 | 1.68 | 0.88 - 2.47 | 3.76E-05 |
| rs612827 | 5 | 3984290 | A | 1.67 | 0.87 - 2.46 | 4.05E-05 | 1.68 | 0.88 - 2.47 | 3.76E-05 |
| rs10466015 | 10 | 71382005 | A | -2.49 | -3.65 - -1.33 | 2.67E-05 | -2.45 | -3.61 - -1.29 | 3.82E-05 |
| rs1543254 | 2 | 99712636 | T | 1.58 | 0.84 - 2.32 | 3.01E-05 | 1.56 | 0.82 - 2.30 | 3.87E-05 |
| rs2309723 | 2 | 99713396 | T | 1.58 | 0.84 - 2.32 | 3.01E-05 | 1.56 | 0.82 - 2.30 | 3.87E-05 |
| rs620703 | 5 | 3997907 | G | 1.66 | 0.87 - 2.46 | 4.18E-05 | 1.68 | 0.88 - 2.47 | 3.87E-05 |
| rs1343548 | 1 | 163481730 | G | -1.58 | -2.36 - -0.80 | 6.81E-05 | -1.64 | -2.42 - -0.86 | 3.89E-05 |
| rs6665694 | 1 | 163474499 | T | -1.58 | -2.36 - -0.80 | 6.81E-05 | -1.64 | -2.42 - -0.86 | 3.89E-05 |
| rs12702926 | 7 | 1564360 | T | 1.74 | 0.93 - 2.55 | 2.77E-05 | 1.71 | 0.90 - 2.52 | 3.95E-05 |
| rs4941939 | 13 | 39150190 | T | -1.56 | -2.32 - -0.80 | 5.64E-05 | -1.60 | -2.36 - -0.84 | 4.00E-05 |
| rs6859778 | 5 | 4027384 | C | 1.62 | 0.84 - 2.40 | 4.98E-05 | 1.65 | 0.86 - 2.43 | 4.04E-05 |
| rs11710551 | 3 | 186041762 | C | 1.87 | 0.98 - 2.77 | 4.36E-05 | 1.88 | 0.98 - 2.78 | 4.14E-05 |
| rs16859344 | 3 | 186043663 | C | 1.87 | 0.98 - 2.77 | 4.36E-05 | 1.88 | 0.98 - 2.78 | 4.14E-05 |
| rs2305240 | 3 | 186049733 | A | 1.87 | 0.98 - 2.77 | 4.36E-05 | 1.88 | 0.98 - 2.78 | 4.14E-05 |
| rs1394342 | 4 | 90440548 | C | -1.64 | -2.45 - -0.83 | 7.29E-05 | -1.70 | -2.51 - -0.89 | 4.14E-05 |
| rs1814762 | 4 | 90442368 | C | -1.64 | -2.45 - -0.83 | 7.29E-05 | -1.70 | -2.51 - -0.89 | 4.14E-05 |
| rs10513799 | 3 | 186032233 | A | 1.87 | 0.98 - 2.77 | 4.38E-05 | 1.88 | 0.98 - 2.78 | 4.16E-05 |
| rs7967863 | 12 | 18839586 | A | -2.67 | -3.97 - -1.38 | 5.27E-05 | -2.71 | -4.01 - -1.42 | 4.16E-05 |
| rs695665 | 22 | 24576287 | C | -2.17 | -3.19 - -1.14 | 3.55E-05 | -2.15 | -3.18 - -1.13 | 4.16E-05 |
| rs6897591 | 5 | 4022857 | T | 1.62 | 0.84 - 2.40 | 5.16E-05 | 1.65 | 0.86 - 2.43 | 4.19E-05 |
| rs4941941 | 13 | 39166279 | G | -1.56 | -2.32 - -0.80 | 5.91E-05 | -1.59 | -2.35 - -0.83 | 4.19E-05 |
| rs7332791 | 13 | 39189145 | T | -1.56 | -2.32 - -0.80 | 5.91E-05 | -1.59 | -2.35 - -0.83 | 4.19E-05 |
| rs9315725 | 13 | 39165806 | C | -1.56 | -2.32 - -0.80 | 5.91E-05 | -1.59 | -2.35 - -0.83 | 4.19E-05 |
| rs9548880 | 13 | 39160246 | T | -1.56 | -2.32 - -0.80 | 5.91E-05 | -1.59 | -2.35 - -0.83 | 4.19E-05 |
| rs1346946 | 4 | 90437334 | T | -1.64 | -2.44 - -0.83 | 7.38E-05 | -1.70 | -2.50 - -0.89 | 4.22E-05 |
| rs7687888 | 4 | 90435770 | T | -1.64 | -2.44 - -0.83 | 7.38E-05 | -1.70 | -2.50 - -0.89 | 4.22E-05 |
| rs10197179 | 2 | 29326440 | G | 2.68 | 1.39 - 3.97 | 4.69E-05 | 2.70 | 1.41 - 3.99 | 4.23E-05 |
| rs9527870 | 13 | 58219208 | T | -1.87 | -2.74 - -1.00 | 2.77E-05 | -1.82 | -2.70 - -0.95 | 4.25E-05 |
| rs4142370 | 13 | 39123469 | C | -1.55 | -2.31 - -0.79 | 6.19E-05 | -1.59 | -2.35 - -0.83 | 4.35E-05 |
| rs4941936 | 13 | 39126333 | C | -1.55 | -2.31 - -0.79 | 6.19E-05 | -1.59 | -2.35 - -0.83 | 4.35E-05 |
| rs4941937 | 13 | 39126457 | A | -1.55 | -2.31 - -0.79 | 6.19E-05 | -1.59 | -2.35 - -0.83 | 4.35E-05 |
| rs4941938 | 13 | 39126805 | C | -1.55 | -2.31 - -0.79 | 6.19E-05 | -1.59 | -2.35 - -0.83 | 4.35E-05 |
| rs4943686 | 13 | 39126510 | T | -1.55 | -2.31 - -0.79 | 6.19E-05 | -1.59 | -2.35 - -0.83 | 4.35E-05 |
| rs9532408 | 13 | 39125090 | A | -1.55 | -2.31 - -0.79 | 6.19E-05 | -1.59 | -2.35 - -0.83 | 4.35E-05 |
| rs9532411 | 13 | 39126141 | T | -1.55 | -2.31 - -0.79 | 6.19E-05 | -1.59 | -2.35 - -0.83 | 4.35E-05 |
| rs9548857 | 13 | 39122116 | G | -1.55 | -2.31 - -0.79 | 6.19E-05 | -1.59 | -2.35 - -0.83 | 4.35E-05 |
| rs9548859 | 13 | 39122527 | C | -1.55 | -2.31 - -0.79 | 6.19E-05 | -1.59 | -2.35 - -0.83 | 4.35E-05 |
| rs9548862 | 13 | 39124900 | G | -1.55 | -2.31 - -0.79 | 6.19E-05 | -1.59 | -2.35 - -0.83 | 4.35E-05 |
| rs9548863 | 13 | 39125353 | G | -1.55 | -2.31 - -0.79 | 6.19E-05 | -1.59 | -2.35 - -0.83 | 4.35E-05 |
| rs9548865 | 13 | 39127368 | A | -1.55 | -2.31 - -0.79 | 6.19E-05 | -1.59 | -2.35 - -0.83 | 4.35E-05 |
| rs9576881 | 13 | 39127121 | C | -1.55 | -2.31 - -0.79 | 6.19E-05 | -1.59 | -2.35 - -0.83 | 4.35E-05 |
| rs9576882 | 13 | 39127137 | C | -1.55 | -2.31 - -0.79 | 6.19E-05 | -1.59 | -2.35 - -0.83 | 4.35E-05 |
| rs12519434 | 5 | 4029212 | G | 1.61 | 0.83 - 2.39 | 5.47E-05 | 1.64 | 0.85 - 2.43 | 4.43E-05 |
| rs17669326 | 10 | 27017942 | C | -1.84 | -2.71 - -0.96 | 3.75E-05 | -1.82 | -2.69 - -0.95 | 4.48E-05 |
| rs17055484 | 13 | 58274144 | C | -1.62 | -2.41 - -0.84 | 5.59E-05 | -1.65 | -2.44 - -0.86 | 4.48E-05 |
| rs10087146 | 8 | 19578375 | C | -2.34 | -3.44 - -1.24 | 3.21E-05 | -2.30 | -3.41 - -1.20 | 4.50E-05 |
| rs6880500 | 5 | 4027281 | A | 1.61 | 0.83 - 2.39 | 5.55E-05 | 1.64 | 0.85 - 2.42 | 4.51E-05 |
| rs4943694 | 13 | 39227032 | T | -1.56 | -2.32 - -0.79 | 6.37E-05 | -1.59 | -2.36 - -0.83 | 4.52E-05 |
| rs9548901 | 13 | 39201194 | T | -1.56 | -2.32 - -0.79 | 6.37E-05 | -1.59 | -2.36 - -0.83 | 4.52E-05 |
| rs1400794 | 5 | 4027744 | C | 1.61 | 0.83 - 2.39 | 5.60E-05 | 1.64 | 0.85 - 2.42 | 4.52E-05 |
| rs6563734 | 13 | 39116271 | A | -1.55 | -2.30 - -0.79 | 6.47E-05 | -1.58 | -2.34 - -0.82 | 4.55E-05 |
| rs7139796 | 13 | 39115841 | A | -1.55 | -2.30 - -0.79 | 6.47E-05 | -1.58 | -2.34 - -0.82 | 4.55E-05 |
| rs7990283 | 13 | 39113956 | A | -1.56 | -2.32 - -0.80 | 5.68E-05 | -1.58 | -2.34 - -0.82 | 4.55E-05 |
| rs9548852 | 13 | 39113741 | C | -1.56 | -2.32 - -0.80 | 5.68E-05 | -1.58 | -2.34 - -0.82 | 4.55E-05 |
| rs9548854 | 13 | 39116954 | A | -1.55 | -2.30 - -0.79 | 6.47E-05 | -1.58 | -2.34 - -0.82 | 4.55E-05 |
| rs12567432 | 1 | 98419459 | A | -1.78 | -2.64 - -0.92 | 5.11E-05 | -1.79 | -2.65 - -0.93 | 4.55E-05 |
| rs1028672 | 13 | 39118387 | A | -1.54 | -2.30 - -0.79 | 6.64E-05 | -1.58 | -2.34 - -0.82 | 4.67E-05 |
| rs725656 | 3 | 186008902 | T | 1.87 | 0.97 - 2.77 | 4.99E-05 | 1.88 | 0.97 - 2.78 | 4.67E-05 |
| rs7325655 | 13 | 39120462 | T | -1.54 | -2.30 - -0.78 | 6.80E-05 | -1.58 | -2.34 - -0.82 | 4.79E-05 |
| rs10041342 | 5 | 4028278 | G | 1.60 | 0.82 - 2.39 | 5.97E-05 | 1.63 | 0.85 - 2.42 | 4.83E-05 |
| rs16871662 | 5 | 3436080 | A | -3.12 | -4.64 - -1.61 | 5.51E-05 | -3.14 | -4.66 - -1.63 | 4.89E-05 |
| rs6712691 | 2 | 29323158 | C | 2.68 | 1.38 - 3.97 | 5.61E-05 | 2.70 | 1.40 - 4.00 | 4.98E-05 |
| rs17705707 | 13 | 58798619 | C | -3.21 | -4.76 - -1.67 | 4.87E-05 | -3.21 | -4.76 - -1.66 | 5.08E-05 |
| rs4451051 | 5 | 4028785 | C | 1.60 | 0.82 - 2.38 | 6.27E-05 | 1.63 | 0.84 - 2.41 | 5.09E-05 |
| rs13184298 | 5 | 4029669 | A | 1.60 | 0.82 - 2.39 | 6.52E-05 | 1.63 | 0.84 - 2.42 | 5.22E-05 |
| rs695748 | 22 | 24580271 | T | -2.02 | -2.99 - -1.05 | 4.71E-05 | -2.01 | -2.99 - -1.04 | 5.30E-05 |
| rs662616 | 13 | 78593828 | C | 2.74 | 1.40 - 4.09 | 6.35E-05 | 2.79 | 1.44 - 4.13 | 5.32E-05 |
| rs13220892 | 6 | 137253181 | G | 2.25 | 1.18 - 3.33 | 4.33E-05 | 2.23 | 1.15 - 3.31 | 5.34E-05 |
| rs1342643 | 6 | 137247665 | C | 2.25 | 1.18 - 3.33 | 4.33E-05 | 2.23 | 1.15 - 3.31 | 5.34E-05 |
| rs17175271 | 6 | 137243692 | A | 2.25 | 1.18 - 3.33 | 4.33E-05 | 2.23 | 1.15 - 3.31 | 5.34E-05 |
| rs4943696 | 13 | 39233127 | C | -1.54 | -2.30 - -0.78 | 7.55E-05 | -1.57 | -2.33 - -0.81 | 5.45E-05 |
| rs11059109 | 12 | 126462910 | G | -1.55 | -2.32 - -0.79 | 7.43E-05 | -1.58 | -2.35 - -0.81 | 5.47E-05 |
| rs1402328 | 12 | 126463932 | C | -1.55 | -2.32 - -0.79 | 7.43E-05 | -1.58 | -2.35 - -0.81 | 5.47E-05 |
| rs1402329 | 12 | 126464037 | C | -1.55 | -2.32 - -0.79 | 7.43E-05 | -1.58 | -2.35 - -0.81 | 5.47E-05 |
| rs7299617 | 12 | 126463716 | C | -1.55 | -2.32 - -0.79 | 7.43E-05 | -1.58 | -2.35 - -0.81 | 5.47E-05 |
| rs10780008 | 3 | 21141414 | G | 1.55 | 0.81 - 2.30 | 4.55E-05 | 1.54 | 0.79 - 2.29 | 5.55E-05 |
| rs6798717 | 3 | 21149801 | C | 1.55 | 0.81 - 2.30 | 4.55E-05 | 1.54 | 0.79 - 2.29 | 5.55E-05 |
| rs16914624 | 12 | 18837472 | C | -2.62 | -3.91 - -1.33 | 7.07E-05 | -2.66 | -3.95 - -1.37 | 5.59E-05 |
| rs16914628 | 12 | 18837791 | C | -2.62 | -3.91 - -1.33 | 7.07E-05 | -2.66 | -3.95 - -1.37 | 5.59E-05 |
| rs7954279 | 12 | 18839434 | C | -2.62 | -3.91 - -1.33 | 7.07E-05 | -2.66 | -3.95 - -1.37 | 5.59E-05 |
| rs13013549 | 2 | 235004322 | G | -1.62 | -2.40 - -0.84 | 4.58E-05 | -1.61 | -2.39 - -0.83 | 5.70E-05 |
| rs6708267 | 2 | 99695363 | C | 1.79 | 0.93 - 2.66 | 4.86E-05 | 1.78 | 0.91 - 2.65 | 5.72E-05 |
| rs6058473 | 20 | 29891075 | A | -2.98 | -4.44 - -1.52 | 6.76E-05 | -3.02 | -4.49 - -1.55 | 5.84E-05 |
| rs10513515 | 3 | 158701927 | C | 2.54 | 1.28 - 3.80 | 8.34E-05 | 2.60 | 1.34 - 3.87 | 5.91E-05 |
| rs501755 | 11 | 115729831 | T | -2.13 | -3.17 - -1.09 | 6.32E-05 | -2.14 | -3.18 - -1.10 | 6.00E-05 |
| rs9538391 | 13 | 58819712 | G | -2.17 | -3.23 - -1.11 | 6.12E-05 | -2.17 | -3.23 - -1.11 | 6.03E-05 |
| rs1146890 | 13 | 75802036 | C | -1.52 | -2.27 - -0.77 | 6.85E-05 | -1.54 | -2.29 - -0.79 | 6.10E-05 |
| rs12434689 | 14 | 78244120 | C | -2.72 | -4.05 - -1.38 | 6.83E-05 | -2.74 | -4.08 - -1.41 | 6.13E-05 |
| rs4388481 | 8 | 136875087 | G | 1.60 | 0.83 - 2.37 | 4.95E-05 | 1.58 | 0.81 - 2.36 | 6.26E-05 |
| rs867859 | 17 | 42653407 | A | -1.75 | -2.63 - -0.87 | 9.77E-05 | -1.80 | -2.68 - -0.92 | 6.29E-05 |
| rs10805105 | 4 | 42549929 | A | -1.55 | -2.32 - -0.78 | 8.17E-05 | -1.58 | -2.36 - -0.81 | 6.33E-05 |
| rs1036745 | 13 | 78499702 | C | 2.72 | 1.37 - 4.08 | 8.30E-05 | 2.78 | 1.42 - 4.14 | 6.34E-05 |
| rs1036746 | 13 | 78499922 | C | 2.72 | 1.37 - 4.08 | 8.30E-05 | 2.78 | 1.42 - 4.14 | 6.34E-05 |
| rs11149122 | 13 | 78496575 | T | 2.72 | 1.37 - 4.08 | 8.30E-05 | 2.78 | 1.42 - 4.14 | 6.34E-05 |
| rs1319622 | 13 | 78500423 | G | 2.72 | 1.37 - 4.08 | 8.30E-05 | 2.78 | 1.42 - 4.14 | 6.34E-05 |
| rs1372180 | 13 | 78495911 | C | 2.72 | 1.37 - 4.08 | 8.30E-05 | 2.78 | 1.42 - 4.14 | 6.34E-05 |
| rs1372181 | 13 | 78495987 | C | 2.72 | 1.37 - 4.08 | 8.30E-05 | 2.78 | 1.42 - 4.14 | 6.34E-05 |
| rs1372182 | 13 | 78501097 | C | 2.72 | 1.37 - 4.08 | 8.30E-05 | 2.78 | 1.42 - 4.14 | 6.34E-05 |
| rs1441257 | 13 | 78496663 | G | 2.72 | 1.37 - 4.08 | 8.30E-05 | 2.78 | 1.42 - 4.14 | 6.34E-05 |
| rs1811440 | 13 | 78499087 | A | 2.72 | 1.37 - 4.08 | 8.30E-05 | 2.78 | 1.42 - 4.14 | 6.34E-05 |
| rs1822152 | 13 | 78500500 | C | 2.72 | 1.37 - 4.08 | 8.30E-05 | 2.78 | 1.42 - 4.14 | 6.34E-05 |
| rs1975416 | 13 | 78497553 | T | 2.72 | 1.37 - 4.08 | 8.30E-05 | 2.78 | 1.42 - 4.14 | 6.34E-05 |
| rs2044011 | 13 | 78499521 | C | 2.72 | 1.37 - 4.08 | 8.30E-05 | 2.78 | 1.42 - 4.14 | 6.34E-05 |
| rs2119542 | 13 | 78498577 | A | 2.72 | 1.37 - 4.08 | 8.30E-05 | 2.78 | 1.42 - 4.14 | 6.34E-05 |
| rs2119543 | 13 | 78498638 | A | 2.72 | 1.37 - 4.08 | 8.30E-05 | 2.78 | 1.42 - 4.14 | 6.34E-05 |
| rs7991135 | 13 | 78497815 | C | 2.72 | 1.37 - 4.08 | 8.30E-05 | 2.78 | 1.42 - 4.14 | 6.34E-05 |
| rs7991177 | 13 | 78497916 | A | 2.72 | 1.37 - 4.08 | 8.30E-05 | 2.78 | 1.42 - 4.14 | 6.34E-05 |
| rs920533 | 13 | 78499293 | C | 2.72 | 1.37 - 4.08 | 8.30E-05 | 2.78 | 1.42 - 4.14 | 6.34E-05 |
| rs9544969 | 13 | 78496317 | C | 2.72 | 1.37 - 4.08 | 8.30E-05 | 2.78 | 1.42 - 4.14 | 6.34E-05 |
| rs9544970 | 13 | 78501883 | A | 2.72 | 1.37 - 4.08 | 8.30E-05 | 2.78 | 1.42 - 4.14 | 6.34E-05 |
| rs9601159 | 13 | 78496546 | G | 2.72 | 1.37 - 4.08 | 8.30E-05 | 2.78 | 1.42 - 4.14 | 6.34E-05 |
| rs984909 | 13 | 78500274 | G | 2.72 | 1.37 - 4.08 | 8.30E-05 | 2.78 | 1.42 - 4.14 | 6.34E-05 |
| rs7379863 | 5 | 3424714 | T | -3.10 | -4.62 - -1.57 | 7.16E-05 | -3.12 | -4.64 - -1.59 | 6.37E-05 |
| rs2008931 | 5 | 4007526 | A | 1.61 | 0.82 - 2.41 | 7.04E-05 | 1.62 | 0.83 - 2.42 | 6.49E-05 |
| rs1487288 | 1 | 98427207 | G | -1.75 | -2.61 - -0.88 | 7.55E-05 | -1.76 | -2.63 - -0.90 | 6.69E-05 |
| rs2748236 | 22 | 24575833 | A | -2.01 | -2.98 - -1.03 | 5.96E-05 | -2.00 | -2.98 - -1.02 | 6.69E-05 |
| rs2859410 | 22 | 24575987 | T | -2.01 | -2.98 - -1.03 | 5.96E-05 | -2.00 | -2.98 - -1.02 | 6.69E-05 |
| rs2859412 | 22 | 24576224 | T | -2.01 | -2.98 - -1.03 | 5.96E-05 | -2.00 | -2.98 - -1.02 | 6.69E-05 |
| rs1968652 | 11 | 131738600 | C | -1.58 | -2.36 - -0.79 | 8.58E-05 | -1.60 | -2.39 - -0.82 | 6.69E-05 |
| rs11907114 | 20 | 53862354 | A | -1.63 | -2.41 - -0.84 | 5.10E-05 | -1.60 | -2.38 - -0.81 | 6.75E-05 |
| rs11051743 | 12 | 32084180 | G | -1.72 | -2.54 - -0.90 | 3.96E-05 | -1.67 | -2.48 - -0.85 | 6.80E-05 |
| rs11051744 | 12 | 32084316 | A | -1.72 | -2.54 - -0.90 | 3.96E-05 | -1.67 | -2.48 - -0.85 | 6.80E-05 |
| rs4931602 | 12 | 32084045 | A | -1.72 | -2.54 - -0.90 | 3.96E-05 | -1.67 | -2.48 - -0.85 | 6.80E-05 |
| rs4931603 | 12 | 32084064 | T | -1.72 | -2.54 - -0.90 | 3.96E-05 | -1.67 | -2.48 - -0.85 | 6.80E-05 |
| rs12479674 | 20 | 29460755 | T | -3.17 | -4.73 - -1.60 | 7.42E-05 | -3.20 | -4.78 - -1.63 | 6.85E-05 |
| rs17248462 | 20 | 29419108 | A | -3.17 | -4.73 - -1.60 | 7.42E-05 | -3.20 | -4.78 - -1.63 | 6.85E-05 |
| rs17249107 | 20 | 29445461 | A | -3.17 | -4.73 - -1.60 | 7.42E-05 | -3.20 | -4.78 - -1.63 | 6.85E-05 |
| rs12146751 | 12 | 32084823 | A | -1.72 | -2.53 - -0.90 | 4.05E-05 | -1.66 | -2.48 - -0.85 | 6.94E-05 |
| rs7677982 | 4 | 14825437 | T | -2.05 | -3.02 - -1.07 | 3.96E-05 | -1.99 | -2.97 - -1.01 | 7.01E-05 |
| rs12643405 | 4 | 42553180 | G | -1.54 | -2.31 - -0.77 | 9.03E-05 | -1.57 | -2.34 - -0.80 | 7.02E-05 |
| rs12645568 | 4 | 42553499 | T | -1.54 | -2.31 - -0.77 | 9.03E-05 | -1.57 | -2.34 - -0.80 | 7.02E-05 |
| rs1450920 | 4 | 42543117 | A | -1.54 | -2.31 - -0.77 | 9.03E-05 | -1.57 | -2.34 - -0.80 | 7.02E-05 |
| rs1450921 | 4 | 42542840 | A | -1.54 | -2.31 - -0.77 | 9.03E-05 | -1.57 | -2.34 - -0.80 | 7.02E-05 |
| rs1901230 | 4 | 42547098 | T | -1.54 | -2.31 - -0.77 | 9.03E-05 | -1.57 | -2.34 - -0.80 | 7.02E-05 |
| rs1993059 | 4 | 42551651 | A | -1.54 | -2.31 - -0.77 | 9.03E-05 | -1.57 | -2.34 - -0.80 | 7.02E-05 |
| rs1993060 | 4 | 42551932 | A | -1.54 | -2.31 - -0.77 | 9.03E-05 | -1.57 | -2.34 - -0.80 | 7.02E-05 |
| rs1993061 | 4 | 42552000 | C | -1.54 | -2.31 - -0.77 | 9.03E-05 | -1.57 | -2.34 - -0.80 | 7.02E-05 |
| rs4479742 | 4 | 42552184 | G | -1.54 | -2.31 - -0.77 | 9.03E-05 | -1.57 | -2.34 - -0.80 | 7.02E-05 |
| rs6817143 | 4 | 42545299 | G | -1.54 | -2.31 - -0.77 | 9.03E-05 | -1.57 | -2.34 - -0.80 | 7.02E-05 |
| rs9548919 | 13 | 39221451 | A | -1.53 | -2.29 - -0.76 | 9.83E-05 | -1.56 | -2.33 - -0.79 | 7.11E-05 |
| rs568306 | 11 | 115728985 | A | -2.10 | -3.15 - -1.06 | 7.58E-05 | -2.11 | -3.16 - -1.07 | 7.22E-05 |
| rs10487038 | 7 | 88272981 | C | 1.65 | 0.84 - 2.46 | 7.15E-05 | 1.65 | 0.84 - 2.47 | 7.30E-05 |
| rs12648708 | 4 | 42559942 | G | -1.54 | -2.31 - -0.77 | 9.40E-05 | -1.57 | -2.34 - -0.79 | 7.31E-05 |
| rs10852143 | 15 | 89423285 | C | 1.59 | 0.81 - 2.36 | 6.97E-05 | 1.58 | 0.80 - 2.36 | 7.32E-05 |
| rs10852144 | 15 | 89423408 | G | 1.59 | 0.81 - 2.36 | 6.97E-05 | 1.58 | 0.80 - 2.36 | 7.32E-05 |
| rs7960035 | 12 | 126462489 | C | -1.52 | -2.29 - -0.76 | 9.86E-05 | -1.55 | -2.32 - -0.79 | 7.37E-05 |
| rs17726834 | 16 | 77659614 | T | -2.71 | -4.00 - -1.43 | 3.73E-05 | -2.62 | -3.91 - -1.32 | 7.45E-05 |
| rs2865697 | 15 | 77487119 | T | 2.77 | 1.39 - 4.14 | 8.18E-05 | 2.78 | 1.41 - 4.16 | 7.49E-05 |
| rs2903061 | 15 | 77486804 | T | 2.77 | 1.39 - 4.14 | 8.18E-05 | 2.78 | 1.41 - 4.16 | 7.49E-05 |
| rs2903062 | 15 | 77486898 | G | 2.77 | 1.39 - 4.14 | 8.18E-05 | 2.78 | 1.41 - 4.16 | 7.49E-05 |
| rs1943418 | 18 | 55630508 | T | -1.81 | -2.72 - -0.90 | 9.84E-05 | -1.84 | -2.76 - -0.93 | 7.55E-05 |
| rs4940906 | 18 | 55628971 | C | -1.81 | -2.72 - -0.90 | 9.84E-05 | -1.84 | -2.76 - -0.93 | 7.55E-05 |
| rs7340698 | 3 | 186060611 | G | 1.81 | 0.91 - 2.70 | 8.05E-05 | 1.82 | 0.92 - 2.72 | 7.58E-05 |
| rs10178628 | 2 | 29324924 | G | 2.62 | 1.31 - 3.93 | 8.63E-05 | 2.64 | 1.34 - 3.95 | 7.62E-05 |
| rs11140707 | 9 | 86440010 | A | 1.63 | 0.84 - 2.42 | 5.51E-05 | 1.61 | 0.81 - 2.40 | 7.63E-05 |
| rs2028285 | 9 | 86446553 | T | 1.63 | 0.84 - 2.42 | 5.51E-05 | 1.61 | 0.81 - 2.40 | 7.63E-05 |
| rs10107740 | 8 | 19576744 | C | -2.39 | -3.55 - -1.23 | 5.53E-05 | -2.35 | -3.51 - -1.19 | 7.76E-05 |
| rs12990926 | 2 | 58527059 | G | 1.70 | 0.86 - 2.54 | 7.16E-05 | 1.70 | 0.86 - 2.54 | 7.77E-05 |
| rs4698322 | 4 | 14826949 | T | -2.03 | -3.01 - -1.06 | 4.43E-05 | -1.98 | -2.96 - -1.00 | 7.78E-05 |
| rs564046 | 11 | 115732055 | G | -2.09 | -3.13 - -1.05 | 8.19E-05 | -2.10 | -3.15 - -1.06 | 7.79E-05 |
| rs1401955 | 3 | 119669281 | C | -1.50 | -2.25 - -0.75 | 8.42E-05 | -1.51 | -2.26 - -0.76 | 7.90E-05 |
| rs6838205 | 4 | 14820991 | C | -2.03 | -3.00 - -1.06 | 4.56E-05 | -1.98 | -2.96 - -1.00 | 8.02E-05 |
| rs333110 | 18 | 7186437 | G | 2.23 | 1.13 - 3.34 | 7.44E-05 | 2.23 | 1.12 - 3.34 | 8.12E-05 |
| rs1460524 | 18 | 39645279 | C | -1.53 | -2.28 - -0.79 | 5.81E-05 | -1.50 | -2.25 - -0.76 | 8.34E-05 |
| rs12324967 | 16 | 77659211 | T | -2.64 | -3.90 - -1.38 | 4.04E-05 | -2.54 | -3.80 - -1.28 | 8.36E-05 |
| rs17812397 | 9 | 102083792 | C | 2.08 | 1.04 - 3.11 | 8.41E-05 | 2.08 | 1.05 - 3.12 | 8.42E-05 |
| rs6541770 | 2 | 121692684 | T | 2.21 | 1.12 - 3.29 | 6.80E-05 | 2.18 | 1.10 - 3.26 | 8.43E-05 |
| rs6541772 | 2 | 121693024 | A | 2.21 | 1.12 - 3.29 | 6.80E-05 | 2.18 | 1.10 - 3.26 | 8.43E-05 |
| rs6555200 | 5 | 3422708 | G | -3.05 | -4.58 - -1.52 | 9.77E-05 | -3.07 | -4.60 - -1.54 | 8.80E-05 |
| rs3115356 | 2 | 235000194 | T | -1.58 | -2.37 - -0.80 | 7.27E-05 | -1.57 | -2.36 - -0.79 | 8.94E-05 |
| rs1396426 | 3 | 3392657 | C | 1.60 | 0.80 - 2.39 | 8.79E-05 | 1.59 | 0.80 - 2.39 | 9.19E-05 |
| rs6746134 | 2 | 99750012 | C | 1.78 |  | 7.89E-05 | 1.76 | 0.87 - 2.64 | 9.89E-05 |
