# Supplementary material for: Moderating effect of mode of delivery on the genetics of intelligence: Explorative genome‐wide analyses in ALSPAC
Source: Brain Behav. 2018 Oct 31;8(12):e01144. doi: 10.1002/brb3.1144 (PMC6305932; doi:10.1002/brb3.1144)
Supplement: Supplementary file 17 [file BRB3-8-e01144-s017.docx]

**S3 Table. Top interaction hits (p<1.00E-04) observed for the PIQ scores.**

| Performance IQ | | | | | | | | | |
| --- | --- | --- | --- | --- | --- | --- | --- | --- | --- |
|  | | | | Crude model | | | Adjusted model | | |
| SNP | CHR | BP | Effect allele | β | C.I. | P value | β | C.I. | P value |
| rs705670 | 9 | 137608405 | G | 2.30 | 1.42 - 3.18 | 3.07E-07 | 2.31 | 1.43 - 3.19 | 3.09E-07 |
| rs12552228 | 9 | 27163693 | T | 2.49 | 1.33 - 3.65 | 2.70E-05 | 2.79 | 1.61 - 3.97 | 3.87E-06 |
| rs12554799 | 9 | 27163704 | G | 2.49 | 1.33 - 3.65 | 2.70E-05 | 2.79 | 1.61 - 3.97 | 3.87E-06 |
| rs12553606 | 9 | 27193764 | C | 2.68 | 1.41 - 3.95 | 3.66E-05 | 3.05 | 1.75 - 4.34 | 4.32E-06 |
| rs10858124 | 9 | 137606640 | A | 1.86 | 1.04 - 2.69 | 9.72E-06 | 1.90 | 1.08 - 2.73 | 6.17E-06 |
| rs4714020 | 6 | 36956027 | T | 1.84 | 1.04 - 2.64 | 7.18E-06 | 1.81 | 1.01 - 2.61 | 9.97E-06 |
| rs9357230 | 6 | 36963360 | C | 1.79 | 0.99 - 2.59 | 1.23E-05 | 1.76 | 0.96 - 2.56 | 1.70E-05 |
| rs17800861 | 16 | 9861173 | A | -2.78 | -4.08 - -1.47 | 3.24E-05 | -2.85 | -4.16 - -1.54 | 2.04E-05 |
| rs11979605 | 7 | 11471725 | T | 3.22 | 1.71 - 4.73 | 3.10E-05 | 3.30 | 1.78 - 4.82 | 2.13E-05 |
| rs7807369 | 7 | 11471491 | G | 3.22 | 1.71 - 4.73 | 3.10E-05 | 3.30 | 1.78 - 4.82 | 2.13E-05 |
| rs11170101 | 12 | 51045770 | C | 2.95 | 1.62 - 4.28 | 1.39E-05 | 2.88 | 1.56 - 4.21 | 2.19E-05 |
| rs2647145 | 1 | 17250477 | C | -2.28 | -3.30 - -1.26 | 1.32E-05 | -2.22 | -3.25 - -1.20 | 2.29E-05 |
| rs1025663 | 13 | 111861792 | A | 1.59 | 0.82 - 2.35 | 5.34E-05 | 1.66 | 0.89 - 2.43 | 2.40E-05 |
| rs1550190 | 13 | 111857270 | C | 1.59 | 0.82 - 2.35 | 5.34E-05 | 1.66 | 0.89 - 2.43 | 2.40E-05 |
| rs17164600 | 7 | 11466577 | T | 3.21 | 1.69 - 4.73 | 3.57E-05 | 3.30 | 1.77 - 4.82 | 2.42E-05 |
| rs7803385 | 7 | 11467661 | T | 3.21 | 1.69 - 4.73 | 3.57E-05 | 3.30 | 1.77 - 4.82 | 2.42E-05 |
| rs6912602 | 6 | 36982426 | A | 2.01 | 1.09 - 2.93 | 1.97E-05 | 1.99 | 1.07 - 2.92 | 2.46E-05 |
| rs17747908 | 8 | 1264651 | T | -1.62 | -2.38 - -0.85 | 3.46E-05 | -1.64 | -2.40 - -0.88 | 2.61E-05 |
| rs3009696 | 9 | 80348263 | T | 1.77 | 0.91 - 2.63 | 5.32E-05 | 1.85 | 0.99 - 2.71 | 2.63E-05 |
| rs3998144 | 10 | 71271319 | C | 1.68 | 0.91 - 2.46 | 2.05E-05 | 1.66 | 0.89 - 2.43 | 2.71E-05 |
| rs12204632 | 6 | 129353430 | T | -2.79 | -4.13 - -1.44 | 4.98E-05 | -2.86 | -4.21 - -1.52 | 3.13E-05 |
| rs2195151 | 3 | 16670470 | C | 1.71 | 0.90 - 2.51 | 3.48E-05 | 1.72 | 0.91 - 2.52 | 3.14E-05 |
| rs9451316 | 6 | 90781576 | T | -1.79 | -2.64 - -0.95 | 3.37E-05 | -1.80 | -2.65 - -0.95 | 3.28E-05 |
| rs10830107 | 10 | 129194065 | G | -2.01 | -2.98 - -1.04 | 4.89E-05 | -2.06 | -3.03 - -1.09 | 3.45E-05 |
| rs8058978 | 16 | 9857437 | G | -2.69 | -3.99 - -1.38 | 5.48E-05 | -2.76 | -4.07 - -1.46 | 3.48E-05 |
| rs2871775 | 1 | 17218492 | A | -1.64 | -2.41 - -0.86 | 3.85E-05 | -1.64 | -2.42 - -0.86 | 3.70E-05 |
| rs4751432 | 10 | 129194988 | T | -1.93 | -2.88 - -0.99 | 6.02E-05 | -1.98 | -2.93 - -1.04 | 4.07E-05 |
| rs2065677 | 10 | 129194434 | A | -1.93 | -2.87 - -0.98 | 6.42E-05 | -1.98 | -2.92 - -1.03 | 4.34E-05 |
| rs17819063 | 16 | 52430929 | A | 3.08 | 1.62 - 4.54 | 3.62E-05 | 3.05 | 1.59 - 4.52 | 4.37E-05 |
| rs357953 | 2 | 3758771 | A | 1.68 | 0.89 - 2.47 | 3.13E-05 | 1.65 | 0.86 - 2.45 | 4.46E-05 |
| rs9380614 | 6 | 36927188 | C | 1.88 | 0.99 - 2.77 | 3.60E-05 | 1.86 | 0.97 - 2.76 | 4.60E-05 |
| rs17198100 | 8 | 103998022 | T | -2.59 | -3.82 - -1.35 | 4.20E-05 | -2.57 | -3.81 - -1.33 | 5.15E-05 |
| rs17011704 | 2 | 75833688 | T | -2.55 | -3.80 - -1.29 | 7.28E-05 | -2.60 | -3.85 - -1.34 | 5.48E-05 |
| rs697449 | 9 | 137607780 | T | 2.12 | 1.11 - 3.12 | 3.68E-05 | 2.07 | 1.07 - 3.08 | 5.53E-05 |
| rs783768 | 9 | 137607587 | C | 2.12 | 1.11 - 3.12 | 3.68E-05 | 2.07 | 1.07 - 3.08 | 5.53E-05 |
| rs2115763 | 11 | 111556379 | T | -1.71 | -2.56 - -0.87 | 7.13E-05 | -1.74 | -2.59 - -0.90 | 5.55E-05 |
| rs2043055 | 11 | 111536834 | G | -1.65 | -2.46 - -0.83 | 7.83E-05 | -1.69 | -2.50 - -0.87 | 5.59E-05 |
| rs7106524 | 11 | 111538846 | A | -1.65 | -2.46 - -0.83 | 7.83E-05 | -1.69 | -2.50 - -0.87 | 5.59E-05 |
| rs11751790 | 6 | 36951601 | T | 1.85 | 0.96 - 2.74 | 4.45E-05 | 1.83 | 0.94 - 2.72 | 5.70E-05 |
| rs11756425 | 6 | 36949734 | G | 1.85 | 0.96 - 2.74 | 4.45E-05 | 1.83 | 0.94 - 2.72 | 5.70E-05 |
| rs2071823 | 6 | 36932377 | T | 1.85 | 0.96 - 2.74 | 4.45E-05 | 1.83 | 0.94 - 2.72 | 5.70E-05 |
| rs9394387 | 6 | 36909263 | C | 1.79 | 0.92 - 2.66 | 5.94E-05 | 1.79 | 0.92 - 2.66 | 5.82E-05 |
| rs2071822 | 6 | 36932079 | A | 1.85 | 0.96 - 2.74 | 4.60E-05 | 1.83 | 0.94 - 2.72 | 5.89E-05 |
| rs7749730 | 6 | 90791705 | G | -1.74 | -2.59 - -0.89 | 6.03E-05 | -1.74 | -2.59 - -0.89 | 6.08E-05 |
| rs4723497 | 7 | 36239499 | C | 1.67 | 0.89 - 2.46 | 3.28E-05 | 1.62 | 0.83 - 2.41 | 6.10E-05 |
| rs9444730 | 6 | 90790058 | G | -2.00 | -2.97 - -1.03 | 5.66E-05 | -1.99 | -2.97 - -1.02 | 6.26E-05 |
| rs12486478 | 3 | 166066762 | C | -1.52 | -2.28 - -0.76 | 8.81E-05 | -1.55 | -2.31 - -0.79 | 6.97E-05 |
| rs11126485 | 2 | 75845302 | T | -2.50 | -3.72 - -1.28 | 6.34E-05 | -2.49 | -3.72 - -1.27 | 7.07E-05 |
| rs10751520 | 9 | 133157444 | C | -1.58 | -2.37 - -0.79 | 9.15E-05 | -1.61 | -2.40 - -0.82 | 7.15E-05 |
| rs11762654 | 7 | 36255100 | A | 1.67 | 0.87 - 2.46 | 3.98E-05 | 1.61 | 0.82 - 2.41 | 7.39E-05 |
| rs7780655 | 7 | 36256526 | G | 1.67 | 0.87 - 2.46 | 3.98E-05 | 1.61 | 0.82 - 2.41 | 7.39E-05 |
| rs471172 | 5 | 146266940 | G | 1.56 | 0.81 - 2.30 | 4.48E-05 | 1.52 | 0.77 - 2.26 | 7.45E-05 |
| rs4855268 | 3 | 166063217 | T | -1.50 | -2.26 - -0.75 | 9.95E-05 | -1.53 | -2.29 - -0.77 | 7.72E-05 |
| rs4764412 | 12 | 18693532 | T | -2.44 | -3.64 - -1.24 | 6.51E-05 | -2.42 | -3.62 - -1.22 | 7.83E-05 |
| rs11044233 | 12 | 18688943 | T | -2.44 | -3.63 - -1.24 | 6.68E-05 | -2.42 | -3.62 - -1.22 | 7.92E-05 |
| rs236387 | 6 | 36892305 | C | 1.60 | 0.81 - 2.40 | 8.12E-05 | 1.61 | 0.81 - 2.40 | 7.94E-05 |
| rs9368965 | 6 | 36922083 | G | 1.83 | 0.93 - 2.73 | 6.47E-05 | 1.81 | 0.91 - 2.71 | 8.17E-05 |
| rs9380607 | 6 | 36904111 | G | 1.71 | 0.86 - 2.56 | 8.73E-05 | 1.71 | 0.86 - 2.56 | 8.91E-05 |
| rs11751844 | 6 | 36912639 | C | 1.74 | 0.87 - 2.61 | 9.16E-05 | 1.75 | 0.87 - 2.62 | 8.91E-05 |
| rs11044234 | 12 | 18691012 | T | -2.42 | -3.62 - -1.22 | 7.47E-05 | -2.40 | -3.60 - -1.20 | 8.97E-05 |
| rs4714015 | 6 | 36913583 | C | 1.74 | 0.87 - 2.61 | 9.52E-05 | 1.74 | 0.87 - 2.61 | 9.28E-05 |
| rs6684770 | 1 | 17198542 | A | -1.77 | -2.65 - -0.90 | 7.69E-05 | -1.76 | -2.64 - -0.88 | 9.34E-05 |
| rs6754749 | 2 | 165182659 | T | 1.91 | 0.96 - 2.86 | 8.22E-05 | 1.91 | 0.95 - 2.87 | 9.37E-05 |
| rs13394222 | 2 | 165168448 | C | 1.93 | 0.97 - 2.90 | 8.65E-05 | 1.94 | 0.97 - 2.91 | 9.51E-05 |
| rs16956820 | 15 | 29597533 | T | 2.66 | 1.36 - 3.95 | 5.85E-05 | 2.58 | 1.28 - 3.88 | 9.92E-05 |
