# Supplementary material for: Moderating effect of mode of delivery on the genetics of intelligence: Explorative genome‐wide analyses in ALSPAC
Source: Brain Behav. 2018 Oct 31;8(12):e01144. doi: 10.1002/brb3.1144 (PMC6305932; doi:10.1002/brb3.1144)
Supplement: Supplementary file 18 [file BRB3-8-e01144-s018.docx]

**S4 Table. Top interaction hits (p<1.00E-04) observed for the FSIQ scores.**

| Full scale IQ | | | | | | | | | |
| --- | --- | --- | --- | --- | --- | --- | --- | --- | --- |
|  | | | | Crude model | | | Adjusted model | | |
| SNP | CHR | BP | Effect allele | β | C.I. | P value | β | C.I. | P value |
| rs17800861† | 16 | 9861173 | A | -3.32 | -4.63 - -2.01 | 7.22E-07 | -3.43 | -4.74 - -2.12 | 2.98E-07 |
| rs12552228 | 9 | 27163693 | T | 2.44 | 1.33 - 3.55 | 1.81E-05 | 2.84 | 1.71 - 3.96 | 8.51E-07 |
| rs12554799 | 9 | 27163704 | G | 2.44 | 1.33 - 3.55 | 1.81E-05 | 2.84 | 1.71 - 3.96 | 8.51E-07 |
| rs8058978 | 16 | 9857437 | G | -3.02 | -4.27 - -1.78 | 2.08E-06 | -3.12 | -4.36 - -1.88 | 9.09E-07 |
| rs12553606 | 9 | 27193764 | C | 2.50 | 1.28 - 3.72 | 6.03E-05 | 2.97 | 1.74 - 4.21 | 2.66E-06 |
| rs9812705 | 3 | 166023866 | A | -1.63 | -2.35 - -0.91 | 1.04E-05 | -1.68 | -2.40 - -0.95 | 5.76E-06 |
| rs12486478 | 3 | 166066762 | C | -1.63 | -2.35 - -0.90 | 1.17E-05 | -1.67 | -2.40 - -0.95 | 6.60E-06 |
| rs11146134 | 10 | 133591739 | T | 1.86 | 1.01 - 2.72 | 2.07E-05 | 1.95 | 1.10 - 2.81 | 7.49E-06 |
| rs4422314 | 3 | 166030858 | A | -1.61 | -2.34 - -0.89 | 1.34E-05 | -1.66 | -2.38 - -0.93 | 7.51E-06 |
| rs1495065 | 3 | 166020729 | A | -1.71 | -2.48 - -0.95 | 1.20E-05 | -1.75 | -2.52 - -0.99 | 7.56E-06 |
| rs4560300 | 3 | 166020117 | A | -1.71 | -2.48 - -0.95 | 1.20E-05 | -1.75 | -2.52 - -0.99 | 7.56E-06 |
| rs4855184 | 3 | 166047953 | T | -1.71 | -2.48 - -0.95 | 1.20E-05 | -1.75 | -2.52 - -0.99 | 7.56E-06 |
| rs7085239 | 10 | 133600131 | G | 1.86 | 1.00 - 2.71 | 2.14E-05 | 1.95 | 1.10 - 2.80 | 7.77E-06 |
| rs11591860 | 10 | 133588995 | A | 1.86 | 1.00 - 2.72 | 2.46E-05 | 1.96 | 1.10 - 2.82 | 7.90E-06 |
| rs7077135 | 10 | 133600189 | C | 1.86 | 1.00 - 2.71 | 2.18E-05 | 1.95 | 1.10 - 2.80 | 7.95E-06 |
| rs9451316 | 6 | 90781576 | T | -1.86 | -2.67 - -1.05 | 6.82E-06 | -1.84 | -2.65 - -1.03 | 8.39E-06 |
| rs9842109 | 3 | 166047273 | G | -1.60 | -2.33 - -0.88 | 1.54E-05 | -1.65 | -2.37 - -0.92 | 8.68E-06 |
| rs10087146 | 8 | 19578375 | C | -2.50 | -3.60 - -1.40 | 8.50E-06 | -2.49 | -3.59 - -1.39 | 9.47E-06 |
| rs4855268 | 3 | 166063217 | T | -1.58 | -2.30 - -0.86 | 1.90E-05 | -1.62 | -2.35 - -0.90 | 1.10E-05 |
| rs12770607 | 10 | 133603058 | C | 1.82 | 0.96 - 2.68 | 3.16E-05 | 1.91 | 1.06 - 2.76 | 1.17E-05 |
| rs825885 | 12 | 128976791 | A | -2.08 | -3.00 - -1.16 | 9.34E-06 | -2.05 | -2.97 - -1.14 | 1.21E-05 |
| rs9444730 | 6 | 90790058 | G | -2.11 | -3.04 - -1.18 | 8.92E-06 | -2.07 | -3.00 - -1.14 | 1.29E-05 |
| rs7749730 | 6 | 90791705 | G | -1.84 | -2.65 - -1.03 | 9.73E-06 | -1.81 | -2.62 - -1.00 | 1.31E-05 |
| rs6060973 | 20 | 29875483 | T | -3.11 | -4.53 - -1.69 | 1.89E-05 | -3.16 | -4.59 - -1.74 | 1.42E-05 |
| rs6058471 | 20 | 29883845 | G | -2.91 | -4.28 - -1.55 | 3.00E-05 | -3.01 | -4.37 - -1.64 | 1.75E-05 |
| rs6060980 | 20 | 29883615 | G | -2.91 | -4.28 - -1.55 | 3.00E-05 | -3.01 | -4.37 - -1.64 | 1.75E-05 |
| rs10107740 | 8 | 19576744 | C | -2.54 | -3.70 - -1.38 | 1.80E-05 | -2.52 | -3.68 - -1.36 | 2.06E-05 |
| rs11905334 | 20 | 29888704 | G | -2.88 | -4.24 - -1.52 | 3.57E-05 | -2.97 | -4.33 - -1.60 | 2.08E-05 |
| rs7269370 | 20 | 29886172 | A | -2.88 | -4.24 - -1.52 | 3.57E-05 | -2.97 | -4.33 - -1.60 | 2.08E-05 |
| rs8090812 | 18 | 46535797 | G | -2.27 | -3.31 - -1.23 | 1.94E-05 | -2.27 | -3.31 - -1.23 | 2.12E-05 |
| rs708361 | 12 | 128972729 | A | -1.97 | -2.86 - -1.07 | 1.81E-05 | -1.95 | -2.84 - -1.05 | 2.20E-05 |
| rs2647145 | 1 | 17250477 | C | -2.23 | -3.21 - -1.24 | 1.02E-05 | -2.14 | -3.12 - -1.15 | 2.28E-05 |
| rs9925106 | 16 | 7231269 | T | -1.86 | -2.76 - -0.97 | 4.63E-05 | -1.93 | -2.82 - -1.04 | 2.37E-05 |
| rs705670 | 9 | 137608405 | G | 1.77 | 0.92 - 2.61 | 4.09E-05 | 1.82 | 0.97 - 2.66 | 2.42E-05 |
| rs7646131 | 3 | 166068526 | G | -1.53 | -2.26 - -0.80 | 4.11E-05 | -1.57 | -2.30 - -0.84 | 2.43E-05 |
| rs6730127 | 2 | 121688981 | C | 2.34 | 1.19 - 3.48 | 6.68E-05 | 2.46 | 1.32 - 3.61 | 2.56E-05 |
| rs7701763 | 5 | 154923437 | T | -1.57 | -2.30 - -0.83 | 3.06E-05 | -1.58 | -2.31 - -0.85 | 2.56E-05 |
| rs12479946 | 20 | 29748070 | C | -2.77 | -4.09 - -1.44 | 4.65E-05 | -2.86 | -4.19 - -1.52 | 2.72E-05 |
| rs12480630 | 20 | 29747273 | T | -2.77 | -4.09 - -1.44 | 4.65E-05 | -2.86 | -4.19 - -1.52 | 2.72E-05 |
| rs17253922 | 20 | 29751357 | C | -2.77 | -4.09 - -1.44 | 4.65E-05 | -2.86 | -4.19 - -1.52 | 2.72E-05 |
| rs6060855 | 20 | 29762606 | T | -2.77 | -4.09 - -1.44 | 4.65E-05 | -2.86 | -4.19 - -1.52 | 2.72E-05 |
| rs17691515 | 16 | 20768573 | A | 2.43 | 1.28 - 3.59 | 3.79E-05 | 2.47 | 1.32 - 3.62 | 2.82E-05 |
| rs17747187 | 16 | 20712750 | T | 2.43 | 1.28 - 3.59 | 3.79E-05 | 2.47 | 1.32 - 3.62 | 2.82E-05 |
| rs1394342 | 4 | 90440548 | C | -1.75 | -2.56 - -0.95 | 2.04E-05 | -1.72 | -2.53 - -0.92 | 2.93E-05 |
| rs1814762 | 4 | 90442368 | C | -1.75 | -2.56 - -0.95 | 2.04E-05 | -1.72 | -2.53 - -0.92 | 2.93E-05 |
| rs17381294 | 1 | 98376789 | A | -1.73 | -2.58 - -0.88 | 7.08E-05 | -1.82 | -2.67 - -0.97 | 2.97E-05 |
| rs17381509 | 1 | 98395255 | G | -1.71 | -2.56 - -0.86 | 8.54E-05 | -1.82 | -2.67 - -0.97 | 2.97E-05 |
| rs17381657 | 1 | 98417071 | G | -1.71 | -2.56 - -0.86 | 8.54E-05 | -1.82 | -2.67 - -0.97 | 2.97E-05 |
| rs963852 | 1 | 98401865 | G | -1.71 | -2.56 - -0.86 | 8.54E-05 | -1.82 | -2.67 - -0.97 | 2.97E-05 |
| rs6058316 | 20 | 29719634 | C | -2.75 | -4.08 - -1.42 | 5.20E-05 | -2.84 | -4.17 - -1.51 | 3.07E-05 |
| rs6058381 | 20 | 29739567 | G | -2.75 | -4.08 - -1.42 | 5.20E-05 | -2.84 | -4.17 - -1.51 | 3.07E-05 |
| rs1346946 | 4 | 90437334 | T | -1.75 | -2.55 - -0.94 | 2.17E-05 | -1.72 | -2.52 - -0.91 | 3.13E-05 |
| rs7687888 | 4 | 90435770 | T | -1.75 | -2.55 - -0.94 | 2.17E-05 | -1.72 | -2.52 - -0.91 | 3.13E-05 |
| rs6060477 | 20 | 29700406 | G | -2.74 | -4.07 - -1.41 | 5.33E-05 | -2.83 | -4.16 - -1.50 | 3.15E-05 |
| rs6060489 | 20 | 29701566 | A | -2.74 | -4.07 - -1.41 | 5.33E-05 | -2.83 | -4.16 - -1.50 | 3.15E-05 |
| rs6060531 | 20 | 29707019 | A | -2.74 | -4.07 - -1.41 | 5.33E-05 | -2.83 | -4.16 - -1.50 | 3.15E-05 |
| rs13200132 | 6 | 36943489 | C | -2.90 | -4.32 - -1.47 | 7.05E-05 | -3.03 | -4.45 - -1.60 | 3.20E-05 |
| rs1943418 | 18 | 55630508 | T | -2.01 | -2.91 - -1.11 | 1.25E-05 | -1.92 | -2.83 - -1.02 | 3.37E-05 |
| rs4940906 | 18 | 55628971 | C | -2.01 | -2.91 - -1.11 | 1.25E-05 | -1.92 | -2.83 - -1.02 | 3.37E-05 |
| rs720321 | 18 | 59033630 | A | -2.22 | -3.30 - -1.13 | 6.59E-05 | -2.28 | -3.37 - -1.20 | 3.72E-05 |
| rs12434689 | 14 | 78244120 | C | -2.76 | -4.09 - -1.44 | 4.77E-05 | -2.80 | -4.13 - -1.47 | 3.73E-05 |
| rs6134838 | 20 | 13190328 | G | -2.72 | -4.03 - -1.41 | 5.11E-05 | -2.75 | -4.07 - -1.44 | 4.03E-05 |
| rs729838 | 20 | 29727189 | A | -2.63 | -3.92 - -1.34 | 6.89E-05 | -2.71 | -4.01 - -1.42 | 4.11E-05 |
| rs11167292 | 20 | 29807763 | C | -2.70 | -4.04 - -1.37 | 7.29E-05 | -2.79 | -4.13 - -1.46 | 4.35E-05 |
| rs11907631 | 20 | 29822157 | C | -2.70 | -4.04 - -1.37 | 7.29E-05 | -2.79 | -4.13 - -1.46 | 4.35E-05 |
| rs12481724 | 20 | 29807408 | G | -2.70 | -4.04 - -1.37 | 7.29E-05 | -2.79 | -4.13 - -1.46 | 4.35E-05 |
| rs17339879 | 20 | 29834553 | T | -2.70 | -4.04 - -1.37 | 7.29E-05 | -2.79 | -4.13 - -1.46 | 4.35E-05 |
| rs2182967 | 20 | 29803046 | G | -2.70 | -4.04 - -1.37 | 7.29E-05 | -2.79 | -4.13 - -1.46 | 4.35E-05 |
| rs6058461 | 20 | 29827905 | G | -2.70 | -4.04 - -1.37 | 7.29E-05 | -2.79 | -4.13 - -1.46 | 4.35E-05 |
| rs6060913 | 20 | 29790501 | C | -2.70 | -4.04 - -1.37 | 7.29E-05 | -2.79 | -4.13 - -1.46 | 4.35E-05 |
| rs6060943 | 20 | 29835115 | C | -2.70 | -4.04 - -1.37 | 7.29E-05 | -2.79 | -4.13 - -1.46 | 4.35E-05 |
| rs7270207 | 20 | 29784544 | C | -2.70 | -4.04 - -1.37 | 7.29E-05 | -2.79 | -4.13 - -1.46 | 4.35E-05 |
| rs1431545 | 4 | 90439682 | C | -1.71 | -2.52 - -0.91 | 3.06E-05 | -1.68 | -2.49 - -0.88 | 4.43E-05 |
| rs10491471 | 5 | 63917942 | G | -3.17 | -4.73 - -1.61 | 7.13E-05 | -3.24 | -4.80 - -1.68 | 4.77E-05 |
| rs10858124 | 9 | 137606640 | A | 1.58 | 0.79 - 2.37 | 8.72E-05 | 1.63 | 0.85 - 2.42 | 4.83E-05 |
| rs13193265 | 6 | 37063644 | A | 1.72 | 0.89 - 2.55 | 5.19E-05 | 1.73 | 0.89 - 2.56 | 4.94E-05 |
| rs17455429 | 11 | 13585466 | A | 2.12 | 1.12 - 3.13 | 3.70E-05 | 2.09 | 1.08 - 3.09 | 4.94E-05 |
| rs471172 | 5 | 146266940 | G | 1.55 | 0.84 - 2.27 | 2.21E-05 | 1.48 | 0.77 - 2.20 | 5.10E-05 |
| rs6889756 | 5 | 127332304 | T | 1.94 | 1.01 - 2.86 | 4.20E-05 | 1.91 | 0.99 - 2.84 | 5.11E-05 |
| rs758181 | 5 | 127330661 | A | 1.94 | 1.01 - 2.86 | 4.20E-05 | 1.91 | 0.99 - 2.84 | 5.11E-05 |
| rs7200633 | 16 | 7223937 | G | -1.80 | -2.70 - -0.90 | 9.66E-05 | -1.87 | -2.77 - -0.96 | 5.15E-05 |
| rs6060963 | 20 | 29862025 | C | -2.68 | -4.01 - -1.34 | 8.64E-05 | -2.77 | -4.10 - -1.43 | 5.19E-05 |
| rs6060964 | 20 | 29864318 | A | -2.68 | -4.01 - -1.34 | 8.64E-05 | -2.77 | -4.10 - -1.43 | 5.19E-05 |
| rs6060965 | 20 | 29869759 | G | -2.68 | -4.01 - -1.34 | 8.64E-05 | -2.77 | -4.10 - -1.43 | 5.19E-05 |
| rs11738877 | 5 | 166011087 | G | 1.67 | 0.87 - 2.47 | 4.49E-05 | 1.65 | 0.85 - 2.45 | 5.64E-05 |
| rs6886538 | 5 | 166013039 | T | 1.67 | 0.87 - 2.47 | 4.49E-05 | 1.65 | 0.85 - 2.45 | 5.64E-05 |
| rs11077164 | 16 | 7236885 | C | -1.99 | -2.98 - -0.99 | 9.58E-05 | -2.04 | -3.04 - -1.05 | 5.68E-05 |
| rs7195406 | 16 | 7235516 | A | -1.99 | -2.98 - -0.99 | 9.58E-05 | -2.04 | -3.04 - -1.05 | 5.68E-05 |
| rs11874104 | 18 | 46539639 | A | -2.12 | -3.15 - -1.09 | 5.45E-05 | -2.12 | -3.15 - -1.09 | 5.81E-05 |
| rs11875985 | 18 | 46538246 | T | -2.12 | -3.15 - -1.09 | 5.45E-05 | -2.12 | -3.15 - -1.09 | 5.81E-05 |
| rs12970340 | 18 | 46540743 | A | -2.12 | -3.15 - -1.09 | 5.45E-05 | -2.12 | -3.15 - -1.09 | 5.81E-05 |
| rs17735708 | 18 | 46538172 | C | -2.12 | -3.15 - -1.09 | 5.45E-05 | -2.12 | -3.15 - -1.09 | 5.81E-05 |
| rs1811455 | 18 | 46537647 | C | -2.12 | -3.15 - -1.09 | 5.45E-05 | -2.12 | -3.15 - -1.09 | 5.81E-05 |
| rs708010 | 6 | 37071350 | A | 1.70 | 0.87 - 2.54 | 6.20E-05 | 1.71 | 0.88 - 2.54 | 5.89E-05 |
| rs6060951 | 20 | 29842712 | T | -2.66 | -3.99 - -1.32 | 9.83E-05 | -2.75 | -4.08 - -1.41 | 5.94E-05 |
| rs6659979 | 1 | 179572574 | A | -1.65 | -2.47 - -0.84 | 7.42E-05 | -1.67 | -2.48 - -0.86 | 6.01E-05 |
| rs12480322 | 20 | 29900302 | C | -2.70 | -4.05 - -1.35 | 9.09E-05 | -2.77 | -4.12 - -1.42 | 6.03E-05 |
| rs12480325 | 20 | 29900327 | A | -2.70 | -4.05 - -1.35 | 9.09E-05 | -2.77 | -4.12 - -1.42 | 6.03E-05 |
| rs4244455 | 8 | 19571816 | G | -2.40 | -3.56 - -1.24 | 5.24E-05 | -2.38 | -3.54 - -1.22 | 6.04E-05 |
| rs6058274 | 20 | 29702707 | T | -2.62 | -3.92 - -1.32 | 7.77E-05 | -2.67 | -3.97 - -1.37 | 6.04E-05 |
| rs4714031 | 6 | 37078989 | T | 1.70 | 0.87 - 2.53 | 6.47E-05 | 1.70 | 0.87 - 2.54 | 6.15E-05 |
| rs9318430 | 13 | 75901687 | T | -2.28 | -3.38 - -1.19 | 4.47E-05 | -2.24 | -3.33 - -1.15 | 6.15E-05 |
| rs9565272 | 13 | 75895769 | G | -2.28 | -3.38 - -1.19 | 4.47E-05 | -2.24 | -3.33 - -1.15 | 6.15E-05 |
| rs17619413 | 1 | 237032286 | T | 1.91 | 1.00 - 2.82 | 4.36E-05 | 1.87 | 0.96 - 2.78 | 6.18E-05 |
| rs4707586 | 6 | 90764003 | T | 1.55 | 0.81 - 2.29 | 4.66E-05 | 1.53 | 0.78 - 2.27 | 6.26E-05 |
| rs9344981 | 6 | 90762790 | T | 1.55 | 0.81 - 2.29 | 4.66E-05 | 1.53 | 0.78 - 2.27 | 6.26E-05 |
| rs9353711 | 6 | 90759159 | C | 1.55 | 0.81 - 2.29 | 4.66E-05 | 1.53 | 0.78 - 2.27 | 6.26E-05 |
| rs12525363 | 6 | 37066711 | T | 1.70 | 0.86 - 2.53 | 6.65E-05 | 1.70 | 0.87 - 2.53 | 6.32E-05 |
| rs17164458 | 5 | 127340423 | G | 1.92 | 0.99 - 2.85 | 5.17E-05 | 1.90 | 0.97 - 2.83 | 6.34E-05 |
| rs13251519 | 8 | 141060141 | A | 2.22 | 1.12 - 3.33 | 8.11E-05 | 2.25 | 1.15 - 3.35 | 6.36E-05 |
| rs13279341 | 8 | 141060063 | A | 2.22 | 1.12 - 3.33 | 8.11E-05 | 2.25 | 1.15 - 3.35 | 6.36E-05 |
| rs4557160 | 3 | 166230624 | T | -1.45 | -2.17 - -0.72 | 9.86E-05 | -1.48 | -2.21 - -0.76 | 6.50E-05 |
| rs2883158 | 1 | 237190804 | C | 1.96 | 0.99 - 2.92 | 7.73E-05 | 1.97 | 1.00 - 2.94 | 6.68E-05 |
| rs17569693 | 16 | 9987686 | G | -2.60 | -3.85 - -1.36 | 4.31E-05 | -2.54 | -3.79 - -1.29 | 6.80E-05 |
| rs6060972 | 20 | 29875261 | T | -2.69 | -4.04 - -1.34 | 9.19E-05 | -2.74 | -4.09 - -1.39 | 7.21E-05 |
| rs11044233 | 12 | 18688943 | T | -2.34 | -3.48 - -1.19 | 6.67E-05 | -2.32 | -3.47 - -1.17 | 7.71E-05 |
| rs1396426 | 3 | 3392657 | C | 1.66 | 0.87 - 2.46 | 4.46E-05 | 1.61 | 0.81 - 2.40 | 7.91E-05 |
| rs1367789 | 4 | 90428182 | C | -2.03 | -3.03 - -1.03 | 7.72E-05 | -2.01 | -3.01 - -1.00 | 9.67E-05 |
| rs4714020 | 6 | 36956027 | T | 1.53 | 0.76 - 2.30 | 9.87E-05 | 1.53 | 0.76 - 2.29 | 9.69E-05 |
| rs4764412 | 12 | 18693532 | T | -2.30 | -3.45 - -1.16 | 8.41E-05 | -2.28 | -3.43 - -1.14 | 9.85E-05 |
| rs9951126 | 18 | 55635276 | T | -1.60 | -2.36 - -0.84 | 3.72E-05 | -1.52 | -2.28 - -0.75 | 9.94E-05 |
| rs11873058 | 18 | 46578885 | C | -2.46 | -3.67 - -1.25 | 7.05E-05 | -2.41 | -3.62 - -1.20 | 9.97E-05 |
| rs920784 | 18 | 46572118 | C | -2.46 | -3.67 - -1.25 | 7.05E-05 | -2.41 | -3.62 - -1.20 | 9.97E-05 |

† dominant model
