# Supplementary material for: Moderating effect of mode of delivery on the genetics of intelligence: Explorative genome‐wide analyses in ALSPAC
Source: Brain Behav. 2018 Oct 31;8(12):e01144. doi: 10.1002/brb3.1144 (PMC6305932; doi:10.1002/brb3.1144)
Supplement: Supplementary file 19 [file BRB3-8-e01144-s019.docx]

**Supporting information**

**S1 Fig. ALSPAC data processing.**

**S2 Fig. Distribution of Verbal IQ scores.**

**S3 Fig. Distribution of Performance IQ scores.**

**S4 Fig. Distribution of Full Scale IQ scores.**

**S5 Fig. Regional plot of rs705670.**

Variants are plotted by position on chromosome 9 against observed interaction p-values for PIQ measures in the adjusted model. Local LD structure is reflected by estimated recombination rates from the HapMap CEU population (Utah residents with Northern and Western European ancestry) plotted in red (right side). The colors of the variants surrounding rs705670 reflect their LD (according to pair-wise r^2^ values from the HapMap CEU population). “Genes” refer to protein-coding genes in the presented region. “refGenes” refer to both protein-coding and non-protein-coding genes reflecting the data from RefSeq UCSC tracks. “lincRNAsAllCellTypeTopView” reﬂects the data from lncRNA UCSC tracks in brain tissue.

**S6 Fig. Regional plot of rs1276529.**

Variants are plotted by position on chromosome 6 against observed interaction p-values for VIQ measures in the adjusted model. Local LD structure is reflected by estimated recombination rates from the HapMap CEU population (Utah residents with Northern and Western European ancestry) plotted in red (right side). The colors of the variants surrounding rs1276529 reflect their LD (according to pair-wise r^2^ values from the HapMap CEU population). “Genes” refer to protein-coding genes in the presented region. “refGenes” refer to both protein-coding and non-protein-coding genes reflecting the data from RefSeq UCSC tracks. “lincRNAsAllCellTypeTopView” reﬂects the data from lncRNA UCSC tracks in brain tissue.

**S7 Fig. Interaction plot reflecting a moderating effect of DCS on the correlation between VIQ scores and rs1276529.**

The Y axis represents the VIQ scores while the X axis displays rs1276529 genotypes. Error bars represent the standard error of the VIQ scores.

**S8 Fig. Interaction plot reflecting a moderating effect of DCS on the correlation between PIQ scores and rs705670.**

The Y axis represents the PIQ scores while the X axis displays rs705670 genotypes. Error bars represent the standard error of the PIQ scores.

**S9 Fig. Quantile-quantile plot showing the distribution of p-values for interactions observed for VIQ scores.**

**S10 Fig. Quantile-quantile plot showing the distribution of p-values for interactions observed for PIQ scores.**

**S11 Fig. Quantile-quantile plot showing the distribution of p-values for interactions observed for FSIQ scores.**

**S12 Fig. Manhattan plot showing results of interaction term for VIQ scores.**

**S13 Fig. Manhattan plot showing results of interaction term for PIQ scores.**

**S14 Fig. Manhattan plot showing results of interaction term for FSIQ scores.**

**S1 Table. Correlation between perinatal factors examined in this study.**

**S2 Table. Top interaction hits (p<1.00E-04) observed for the VIQ scores.**

**S3 Table. Top interaction hits (p<1.00E-04) observed for the PIQ scores.**

**S4 Table. Top interaction hits (p<1.00E-04) observed for the FSIQ scores.**
